# Genome organization, virulence genes, and temperature-dependent motility of an emerging pathogen, *Escherichia marmotae*

**DOI:** 10.3389/fmicb.2025.1729604

**Published:** 2025-12-17

**Authors:** Pelumi M. Oladipo, Ali M. Jomaa, Jeffrey H. Withey, Jeffrey L. Ram

**Affiliations:** 1 Department of Physiology, Wayne State University School of Medicine, Detroit, MI, United States; 2 Department of Biochemistry, Microbiology, and Immunology, Wayne State University School of Medicine, Detroit, MI, United States

**Keywords:** antimicrobial resistance, biofilm formation, *Escherichia coli*, *Escherichia marmotae*, motility, RT-qPCR, virulence

## Abstract

**Introduction:**

*Escherichia marmotae* is one of the *Escherichia* cryptic clades that were first isolated from animal feces and environmental waters and has recently emerged as an organism of concern due to its presence in human infections. Although *E. marmotae* cannot be distinguished from *E. coli* by standard clinical tests, its 10% pairwise genomic difference from *E. coli* led us to investigate other phenotypic differences that may be present.

**Methods:**

Bioinformatic software was used to identify the *E. marmotae* pan-genome, antimicrobial and virulence genes, and sequences of genes for motility, biofilm formation, and other phenotypic characteristics. Environmental and clinical isolates of *E. marmotae* were analyzed for antimicrobial sensitivity, and for temperature effects on motility, growth, and biofilm formation, in comparison to *E. coli*. RT-PCR analyzed associated changes in gene expression.

**Results:**

The *E. marmotae* genome consists of >75% core genes, and has many accessory genes, including plasmids and antimicrobial resistance genes. *E. marmotae* is resistant to erythromycin. *E. marmotae* had all genes needed for complete flagellar gene assembly, and phenotypically was motile at 28°C, and much less motile at 37°C. More biofilm formation was observed at 28°C than at 37°C. The expression of motility genes motA and fliA decreased at 37°C in *E. marmotae* compared to *E. coli*.

**Conclusion:**

These temperature-sensitive traits may support environmental persistence and adaptations that may facilitate *E. marmotae* to cause human disease.

## 1 Introduction

*Escherichia marmotae* was initially described as Cryptic Clade V (Walk et al., 2009), the *Escherichia* cryptic clade that diverged the furthest from *Escherichia coli*, exhibiting a 10% pairwise difference from *E. coli* in its whole genome sequence (Liu et al., 2015). Subsequent to its initial discovery in environmental samples, raccoons, birds, and dogs (Walk et al., 2009), Cryptic Clade V strains were isolated from the feces of *Marmota himalaya* in Tibet, characterized with respect to additional phenotypic properties, and named *Escherichia marmotae* (Liu et al., 2015). *E. marmotae* has also been isolated from septicemic patients (Clermont et al., 2011; Lefort et al., 2011; Oladipo et al., 2025), diaphragms of wild boars, and the feces of cows (Ocejo et al., 2020; Binsker et al., 2024), bank voles (Zhurilov et al., 2023), farm healthy hens (Gilroy et al., 2021; Thomson et al., 2022), food (Binsker et al., 2024) and many species of birds (Walk et al., 2009; Sakaguchi et al., 2023; Souguir et al., 2023).

Wild animals are a potential reservoir of *E. marmotae* that could be pathogenic in humans (Liu et al., 2015, 2019). *In vitro* studies to understand the potential virulence of *E. marmotae* observed that *E. marmotae* adheres to and moderately invades human epithelial cells (Liu et al., 2019). Similarly, the *E. marmotae* strain isolated from wild bank vole was shown to adhere to human epithelial cells and red blood cells but was capable of invading a Hep-2 cell line but somewhat less effectively than Salmonella enterica (Zhurilov et al., 2023). Further investigations of the invasive capability of *E. marmotae* are needed.

*E. marmotae* was subsequently described in four isolates from human clinical samples (Sivertsen et al., 2022). As the authors describe, these isolates were first misidentified as *E. coli* using MALDI-TOF MS, but due to a low ranking score, the isolates were subjected to genome sequencing and determined to be *E. marmotae*. The human infections associated with these clinical samples included thoracic spondylodiscitis, pyelonephritis, acute sepsis of unknown origin, and postoperative sepsis. Similarly Sinha et al. (2023), identified a strain of *Escherichia* associated with a urinary tract infection (UTI) that was determined to be *E. marmotae* by 16S rRNA sequencing and a divergent MALDI-TOF MS. Thus, *E. marmotae* and *E. coli* appear to share similar pathogenic traits, similar to extraintestinal pathogenic *E. coli* (ExPEC) such as type I fimbriae, K1 capsule, invasion protein (*ibeA, ibeB, ibeC*) and ompA porin that can colonize sites outside the intestinal tract and cause a range of serious illnesses, including sepsis, meningitis, and urinary tract infections (Sakaguchi et al., 2023; Zhurilov et al., 2023). As recounted here, the identification of *E. marmotae* by conventional laboratory diagnostics (biochemical test and MALDI- TOF MS) has been challenging and when found, it was originally misidentified as *E. coli*, indicating a need for improved diagnostic methods, the subject of another study from our laboratory (Oladipo et al., 2025).

Despite its identification in several cases of human disease, the pathogenicity mechanisms and the association of *E. marmotae* with illnesses among humans and animals remain under-explored. Virulence genes for enterotoxins, adhesion, motility, and biofilms have been detected in both environmental and clinical strains of *E. marmotae* (Liu et al., 2019; Sivertsen et al., 2022; Sakaguchi et al., 2023; Zhurilov et al., 2023; Siddi et al., 2024); however, the roles of these virulence genes in the reported infections is unknown. Motility has an important role in pathogen-host interactions, promoting colonization, adherence, and biofilm formation and maturation (Wood et al., 2006; Duan et al., 2013). Contradictory reports about the motility of *E. marmotae* have been published, with some identifying it as motile (Zhurilov et al., 2023) and others as non-motile (Liu et al., 2015). Biofilm formation, a key factor in many persistent and chronic infections, may contribute to its pathogenicity, with flagellar-mediated motility playing a key role in this process (Wood et al., 2006; Duan et al., 2013; Vestby et al., 2020). Further investigation is needed as motility and biofilm formation could significantly impact the infection process. In this study, we conducted a comparative analysis of the virulome and antimicrobial resistance profile of *E. marmotae* using whole genome sequencing of our strains and of whole genomes available in GenBank, and investigated the motility and biofilm formation of *E. marmotae* compared to *E. coli*. We found that although *E. marmotae* strains have many phenotypic similarities to *E. coli*, some phenotypic and gene expression properties are greatly different, including significant differences in motility and its temperature-dependence, and in biofilm formation, in comparison to *E. coli*.

## 2 Materials and methods

### 2.1 Bacterial isolates

The Ram laboratory previously isolated five strains of *E. marmotae* from aquatic environments and raccoons (Ram et al., 2007). These isolates were archived at −80 ± 2°C in glycerol stocks [in Colilert 18 (IDEXX US) media with 15% glycerol] and have reliably yielded viable subcultures. In addition to these isolates from the Ram laboratory, the present study also investigated strains of *E. marmotae* isolated by others from human sources (Sivertsen et al., 2022), and additional isolates of *E. marmotae* (Cryptic Clade V) and other cryptic clades from the microbial archives of Michigan State University, described originally by Walk et al. (2009). In all cases, representative strains were reisolated from glycerol stocks by streaking on Luria-Bertani (LB) agar and regrowing individual colonies in Colilert 18 medium.

### 2.2 Genome sequencing and data analyses

#### 2.2.1 Illumina whole genome sequencing

For bioinformatic analysis, whole genome sequences of the *E. marmotae* strains in the Ram lab and other genomes of *E. marmotae* uploaded to GenBank (Liu et al., 2015; Ocejo et al., 2020; Sivertsen et al., 2022; Rumball et al., 2023; Zhurilov et al., 2023) were analyzed, as described here, and identified by GenBank accession numbers in Results. Genomic DNA extraction utilized a DNeasy Blood and Tissue kit (Qiagen) according to the manufacturer’s instructions. Sequencing was performed on an Illumina NovaSeq 6000 Sequencer by SeqCenter in Pittsburgh, PA, United States. *De novo* assembly of short reads was performed using Unicycler 0.5.0 (Wick et al., 2017). Assembly statistics were recorded with QUAST 5.2.0 (Gurevich et al., 2013), which measures the quality and completeness of the genome assembly (Supplementary Table 1). Genome annotation was performed using Prokka v1.14.6, a prokaryotic genome annotation software tool (Seemann, 2014). The JSpeciesWS online web server (accessed 2024, version unknown (Richter et al., 2016)^1^ was used to calculate nucleotide level similarities between the *E. marmotae* genomes and representative *E. coli* genomes using the ANIb (Average Nucleotide Identity based on BLAST) algorithm.

#### 2.2.2 Pan-genome analyses and ontology, and species-specific genes

Pan-genome analysis of *E. marmotae* whole genomes investigated their microbial diversity in terms of protein coding, functional domains, and regulatory elements. Eighteen *E. marmotae* genomes, listed in Supplementary Table 2 with their GenBank accession information, were selected to determine a generalizable pan-genome. The genomes listed in Supplementary Table 2 include four from isolates that had been isolated and sequenced by the Ram laboratory (this paper) and fourteen whole genome sequences that had previously been deposited in GenBank. Pan-genome analysis was performed using Roary v3.13.0 (Page et al., 2015; Sitto and Battistuzzi, 2020). Roary, a high-speed stand-alone pan-genome pipeline (Page et al., 2015; Sitto and Battistuzzi, 2020), processes annotated assemblies in GFF3 format to compute the pan-genome, highlighting core and strain-specific genes. The GFF3 format files of 18 genomes of *E. marmotae* were produced using PROKKA. The minimum percentage identity for BLASTp searches was set to 95%. Heatmaps were generated in R, and the gene occurrence curve was produced via ggplot2 using the results of Roary.

#### 2.2.3 Single nucleotide polymorphisms analysis

Single Nucleotide Polymorphisms (SNP) were identified using Snippy v4.5 Seemann (2015), with default parameters. Snippy was used to compare *E. marmotae* genomes to *E. coli sequences*, identifying specific genes with synonymous variations, missense mutations, frameshift mutations, disruptive mutations, and other genomic alterations. The complete genomes of *E. coli* K-12 MG16655 (Genbank accession number CP009685), *E. coli* J86-ST05 (Genbank accession number BHVN01000001), and *E. coli* O157: H7 (Genbank accession number JHLK00000000) were used as reference genomes for WGS alignment to ensure consistency in assembly. We analyzed four *E. marmotae* genomes using Snippy to identify SNPs. The potential functional impact of these SNPs was assessed through SnpEff v4.3T (Cingolani et al., 2012), via Snippy. For genes containing non-synonymous variations, we conducted a functional enrichment analysis using the PANTHER classification system V.14.0 (Mi et al., 2019). Because of the focus of the present study on antibiotic resistance and on motility and biofilm formation, we focus the presentation in the main text on genes involved in those functions, with additional results on other functional groups provided in Supplementary material.

#### 2.2.4 Serotypes and virulence genes

*E. marmotae* genomes were analyzed with SerotypeFinder 2.0 (Joensen et al., 2015) to identify serotypes markers. Abricate V1.0.1 was used to screen the whole genome sequence assemblies (Seemann, 2014) for virulence sequences [Virulence Finder, *E. coli* Virulence Factor (Ecoli-VF)], and Antimicrobial resistance (AMR) genes (Resfinder v4.1, Bortolaia et al., 2020). Identification and functional classification used the Virulence Factors Database (VFDB) of Pathogenic Bacteria^2^ (updated 2024). Antibiotic resistance gene identification utilized the NCBI Bacteria Antimicrobial Resistance Reference Gene Database and the Comprehensive Antimicrobial Resistance Database (CARD v3.2.6) (McArthur et al., 2013), setting the identity threshold at 85% nucleotide identity and classifying the drug category with the database found at https://card.mcmaster.ca/browse. Tools from the Center for Genomic Epidemiology^3^ were used to detect plasmid replicons using PlasmidFinder 2.1 (Carattoli et al., 2014), determine sequence types using MLST 2.0 (Larsen et al., 2012), and identify mobile genetic elements using MobileElementFinder v1.0.3 (Johansson et al., 2021).

### 2.3 Antibiotic susceptibility tests

The Minimum Inhibitory Concentrations (MICs) of antimicrobials in *E. marmotae* were determined using gram-negative Sensititre standard AST plates designed for the Sensititre Antimicrobial Susceptibility System (Trek Diagnostic Systems, OH, United States). Colonies were used to prepare a suspension equivalent to a 0.5 McFarland standard using the Sensititre nephelometer. A disposable 10 μL calibrated pipette was used to transfer the suspension into 10 mL of Sensititre cation-adjusted Mueller-Hinton broth. A Sensititre AIM™ Automated Inoculation Delivery System was used to inoculate 50 μL of the inoculated Mueller-Hinton broth into the AST plates. The plates were incubated at 37°C and read after 18–24 h using the Sensititre Vizion Digital MIC Viewing System linked to a computer. Each isolate was tested in biological triplicate, and for each biological replicate, two independent Senstitre plates were used. The Sensititre gram negative plates include the various antibiotics Amikacin, ampicillin, aztreonam, carbenicillin, cefazolin, cefepime, ceftazidime, ceftolozane, ceftriaxone, ciprofloxacin, doripenem, ertapenem, gentamicin, imipenem, levofloxacin, meropenem, minocycline, nitrofurantoin, piperacillin, tetracycline, tigecycline, tobramycin, and trimethoprim in a range of concentrations.

A broth dilution method was used to test the antibiotic sensitivity of *E. marmotae* to erythromycin, nalidixic acid, kanamycin, and streptomycin. Bacterial suspensions were adjusted to a 0.5 McFarland standard. A volume of 200 μL of the standardized bacterial culture was added to each well of a 24-well microtiter plate containing 2 mL of broth supplemented with Colilert reagent and varying concentrations of antibiotics (ranging from 0.5 to 128 μg/mL, depending on the antibiotic). Each concentration was tested in duplicate. The plates were incubated at 37°C for 18–24 h. Following incubation, bacterial growth was assessed under UV light with a blue coloration indicating β-glucuronidase activity. Each isolate was tested in triplicate using three independently cultured biological replicates, with duplicate wells per concentration as technical replicates. Resistance breakpoints, according to the Clinical and Laboratory Standards Institute (CLSI) (Performance Standards for Antimicrobial Susceptibility Testing. 33rd ed. CLSI supplement M100) (Clinical and Laboratory Standards Institute, 2023., n.d.), were used to interpret the MICs of *E. marmotae*. Susceptibility and resistance were compared to predictions based on genomic bioinformatic analyses.

### 2.4 Motility-related experiments

#### 2.4.1 Motility assays

Bacterial motility was measured in semi-solid (0.25% agar) plates containing LB broth, an environment that allows motile bacteria inoculated into the center of the plate to migrate visibly from the center within 24 h; in comparison, non-motile bacteria remain at the site of inoculation (Kakkanat et al., 2017). To conduct these motility tests, *E. marmotae and E. coli* cultures were grown in LB broth overnight at 37°C with shaking at 180 rpm. Cultures were adjusted to OD_600_ = 0.06–0.1. A sterile stab was used to inoculate the culture into the center of the semi-solid agar plate (100 mm × 15 mm). Plates were incubated 18–24 h at 17, 28, and 37 °C. These three temperatures were chosen to represent a range of environmental (28 °C, the approximate highest Great Lakes water temperatures in our area; 17 °C, to simulate cooler summertime environmental conditions) and host-associated (37 °C, various human and other hosts) conditions. Each isolate was tested in biological triplicate, and each biological replicate was plated in duplicate. The result was recorded photographically, and the diameter of the hazy area (bacteria present around the point of inoculation) was measured. On this assay, typical strains of *E. coli* usually spread over the full 5 cm diameter plate at 37 °C (Abdulkadieva et al., 2022); whereas, *E. coli* in which motility genes have been knocked out typically have bacterial spread diameters of < 0.5 cm. Intermediate spreads can also occur, indicating the presence of motility but less than compared to typical wild-type *E. coli*.

#### 2.4.2 Bacteria growth analysis

Because the interpretation of colony spread in the motility assays could alternatively be due to differences in colony growth, we also assayed cultures for growth at different temperatures by previously established methods (Farshadzadeh et al., 2018; Paksanont et al., 2018). Bacteria strains were incubated at 28°C or 37°C overnight in a shaker/incubator. After adjusting the OD_600_ to 0.06–0.1, 200 μL of cultures were pipetted into 96-well plates in quintuplicate, and growth was monitored using a Biotek Synergy H1 Microplate Reader, measuring absorbance at 600 nm at 60-min intervals over a 24 h period. In a separate series of experiments, the initial exponential growth phase was measured every 10 min over 5 h.

#### 2.4.3 Expression of motility relevant genes

##### 2.4.3.1 RNA extraction

Individual colonies of *E. marmotae* were picked, inoculated into 5 mL of LB broth, and incubated at 28 and 37°C under static conditions for 18 h. After incubation, the overnight culture was diluted 1:100 in fresh LB broth, incubated further, and harvested at an optical density (OD_600_) of 0.5–0.6. The bacteria were pelleted by centrifugation at 5,000 × g for 10 min, and RNA was extracted using an RNeasy kit (Qiagen, Germany). The modifications included adding 700 μL of Buffer RW1 to the RNeasy spin column before adding the DNase I stock solution. All centrifugation steps were performed for 30 s at > 8,000 × g (12,000 rpm). Purified RNA was collected by placing the spin column in a new collection tube and centrifuging for 1 min at > 8,000 × g (14,500 rpm). The integrity of the purified RNA was examined by agarose gel electrophoresis, visualizing the 23S/16S banding pattern (Supplementary Figure 2). RNA concentration was measured using a NanoDrop 2000 spectrophotometer, Thermo Scientific, United States).

##### 2.4.3.2 RT-qPCR

cDNA synthesis was carried out using SuperScript IV Reverse Transcriptase for RT-qPCR (Bio-Rad, United States). One microgram of total RNA was reverse-transcribed in a 20 μL reaction volume, according to the manufacturer’s instructions. We measured the temperature-dependent expression of genes associated with motility and assessed the expression levels of key flagella-associated genes from three classes of flagellar genes:*fliA*, *flhD, fliC, flgM*, and *motA*. Class I (e.g., *flhD*) encodes the master regulator controlling initiation of flagellar transcription; Class II genes (*fliA*, *flgM*) regulate assembly of the basal body–hook structure and modulate σ^∧^28 activity; and Class III genes (*fliC*, *motA*) encode components essential for filament formation and flagellar rotation. These genes were selected as canonical markers representing regulatory, structural, and motor functions within the flagellar hierarchy. All primer pairs were designed using Geneious Prime software (Dotmatics). The primer sequences are shown in Table 1. Only conserved regions of the genes shared with *E. marmotae*, *E. coli*, and other cryptic clades were selected for primer design. The amplification was performed in duplicate in a total volume of 25 μL, containing 12.5 μL of Universal SYBR Green (Bio-Rad), 7.5 μL of nuclease-free water, 2.0 μL of each primer (10 μL), and 1 μL of cDNA. RT-qPCR was performed using the CFX 96 Touch Real-time PCR detection system with Bio-Rad CFX manager software version 3.1. The cycling parameters were as follows: 95°C for 5 min, followed by 30 cycles of 95°C for 30 s, annealing temperature specified in Table 1 for 30 s, and 72°C for 40 s.

**TABLE 1:** Oligonucleotide primers used for RT-qPCR.

Gene	Primer sequencing (5′ 3′)	Annealing temperature
*Adk*	Forward- ATTCTGCTTGGCGCTCCGGG Reverse- CCGTCAACTTTCGCGTATTT Adapted from (Walk et al., 2009)	56°C
*FliA*	Forward- CGTATCCGTGGTGCTATGCT Reverse- TGCTATCACCGTGCTCTTCG	56°C
*Mota*	Forward- CTCGGTACAGTTTTCGGCGG Reverse- TGCCCATCTGTCGCGATTTC	56°C
*Flhd*	Forward- TGCATACCTCCGAGTTGCTGA Reverse- AGATCGTCAACGCGGGAATC	57°C
*fliC*	Forward: CCGTATTCAGGACGCCGACT Reverse: GAGTTACCCGCCTGCTGGAT	56°C
*Flgm E. coli specific*	Forward: GTAAGCACCGTTCAACCGCG Reverse: CGCTTAACGTCACACTGGTGC	57°C
*FlgM E. marmotae specific*	Forward: TGAGTATTGACCGCACCGCT Reverse: GTCGTTTTTGTCGAGCGGGT	57°C

For each condition, five independent biological replicates were performed, and each RT-qPCR was run in technical duplicate and the average Ct value was used for analysis. The *adk* gene was used as the reference for calculating the relative expression levels of other genes. The normalized expression levels were calculated by using the 2^–△Ct^ method, where △ Ct = Ct target gene—Ct reference gene).

#### 2.4.4 Negative stain electron microscopy

Bacterial cultures were grown in LB broth at 37 and 28°C overnight. Cells were washed with PBS and fixed in 2.0% (v/v) glutaraldehyde. Samples were directly applied to 300-mesh Cu grids and allowed to adsorb for 1 min. Grids were washed in distilled water and stained with 2% (w/v) uranyl acetate for visualization on a Zeiss Gemini SEM 300 imaged using a STEM detector.

### 2.5 Biofilm formation

Biofilm formation was measured using a modified protocol developed by O’Toole (2011) in test tubes. Each isolate was cultured on LB agar overnight, and a colony of each isolate was suspended in LB broth and incubated overnight. Bacterial suspensions were adjusted to an optical density of 0.5 McFarland standard at OD_600_, and 2 mL was added to each well in flat-bottomed 24-well sterile cell culture plates, as well as into sterile glass tubes (clear visual documentation of biofilm formation at the air–liquid interface). Following a 48 h incubation period at 28 and 37°C, non-adherent bacteria were removed by washing three times with Phosphate-Buffered Saline (PBS). The remaining adherent bacteria were stained by adding 2 mL of 0.1 crystal violet to each well and incubating at room temperature for 15 min; the plates were then washed three times with PBS and dried. Then, 2 mL of 30% acetic acid was added to each well, and absorbance was measured at OD_590_ using a Biotek Synergy H1 Microplate Reader. Each assay was performed in triplicate, and the experiment was conducted three times on each isolate.

### 2.6 Statistical methods

Statistical analysis was performed using GraphPad Prism (version 10.4.0). Specific statistical tests that were used are described in figure captions. As a general rule, prior to applying parametric statistics, normality tests were conducted, and data were accepted as normally distributed if *P* > 0.05 on the Shapiro-Wilk test. If data were not normal, we applied a transformation [e.g., for gene expression data, data were transformed using the function Y = Log (Y)], and the transformed data was confirmed as normally distributed with the Shapiro-Wilks test prior to applying parametric statistics. Where data lacked a normal distribution due to reaching a maximum posssible value (e.g., motility spread diameter that covered the full plate) non-parametric or mixed methods ANOVA analysis provided by GraphPad was used to determine statistical significance.

## 3 Results

### 3.1 Whole genome sequences

Whole genome sequencing (WGS) of *E. marmotae* and *E. coli* isolates determined that 18 *E. marmotae* strains, including five environmental strains of *E. marmotae* from the Ram Lab (Ram et al., 2007), have > 98% identity to one another but differ on average by 9.4% ± 0.2% (mean ± SD) from three environmental strains of *E. coli*. Supplementary Table 1 provides a summary of the genome assembly metrics (number of contigs; total assembled lengths; etc.) for the newly sequenced *E. marmotae* isolates from the Ram Lab, for which the average assembled length was 4,717,033 bp, a maximum assembled length of 5,173,310 bp and an average GC content of 50.4%. Contig counts ranged from 74 to 408, with N50 values between 177,536 and 330,820 bp, indicating relatively contiguous assemblies. These assembled genome lengths and the comparable values for *E. coli* and the *E. marmotae* genomes downloaded from GenBank are shown in Supplementary Table 2. Supplementary Table 2 also provides metadata describing the environmental sources of the *E. marmotae* strains, as well as accession numbers of the GenBank *E. marmotae* sequences to which they are being compared. Supplementary Table 3 shows the percentage identities between *E. marmotae* and *E. coli* strains, over their whole genomes.

### 3.2 Pan-genome analysis

**FIGURE 1:**
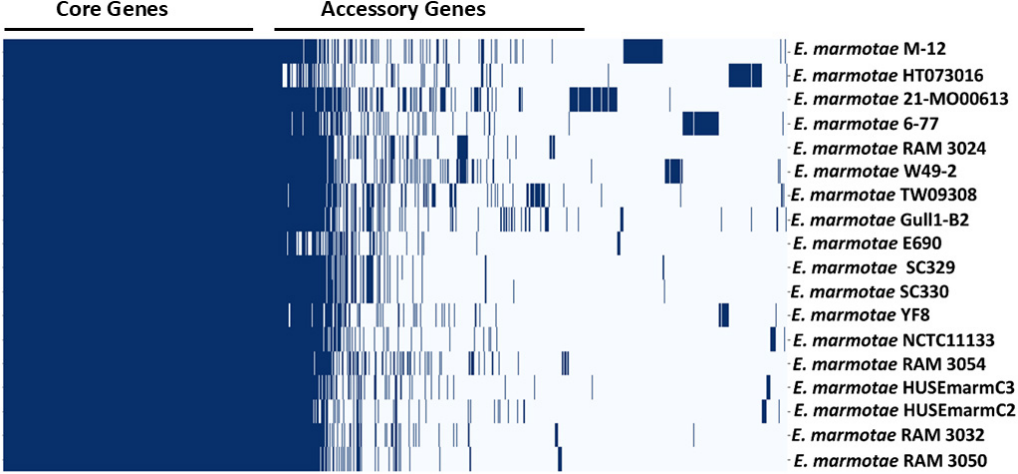
Pan-genome map showing the core and accessory genes, compared among 18 genomes of *E. marmotae*, as listed Supplementary Table 2. The blue bars represent the presence of a gene. A complete listing of all of the pan-genome genes and the number of *E. marmotae* strains in which they appear are listed in Supplementary Table 4.

To begin the functional analysis of the *E. marmotae* whole genomes, pan-genome analysis of 18 *E. marmotae* whole genomes by Roary identified 8,566 gene clusters, which consist of 3,095 core genes, 2,475 accessory genes, and 2,996 strain-specific genes (Figure 1). A gene frequency plot (Supplementary Figure 1) shows an overview of the frequency of genes within the set of 18 whole genomes. Bar 18 shows the number of core genes found in all 18 genomes (3,095 genes; 36.1% of this 18 genome pan-genome), reflecting the unity of within-species function. The core genes accounted for > 75% of the genes in any particular isolate. On the other hand, *E. marmotae* also has 2,996 isolate-specific genes (accounting for 35.0% of the pan-genome) that are unique to individual genomes (Bar 1 in Supplementary Figure 1). Some of these isolate-specific genes are due to mobile elements or plasmids in the whole genome (Supplementary Figure 3). On average, isolate-specific genes account for 4% ± 1% (mean ± SEM) of each isolate’s genome.

Supplementary Table 4 shows the presence and absence matrix of all 18 *E. marmotae* genomes of the pan-genome. As will become clear in subsequent ontology analysis, key components among the core genes of *E. marmotae* are genes coding for the core motility machinery and its regulation, which are consistently present across *E. marmotae* genomes. This core set includes essential motility genes, highlighted in Supplementary Table 4 in yellow, such as *flhD, flhC, motA, motB, fliA, fliC, fliE, fliF, fliG, fliH, fliK, fliL, fliM, fliN, fliO, fliP, fliQ, fliR, fliS, fliZ, cheA, tsr, tar, cheZ, flgA, flgB, flgC, flgD, flgE, flgF through flgN, and flhA* through *flhD*. Additionally, genes implicated in biofilm formation (highlighted in blue in Supplementary Table 4)—such as *luxS*, *fimH*, the *csgABCDE* curli proteins, and type I fimbriae genes (*fimA*, *fimC*, *fimD*, *fimG*, *fimH*)—are likewise present across *E. marmotae* genomes. Many of these genes are also revealed as part of the “virulome” of *E. marmotae*, as further analyzed in section 3.4. Genes that potentially mediate antibiotic resistance are also found in Supplementary Table 4 and further analyzed in this paper in section 3.5.

### 3.3 Variant calls: single nucleotide polymorphism

**FIGURE 2:**
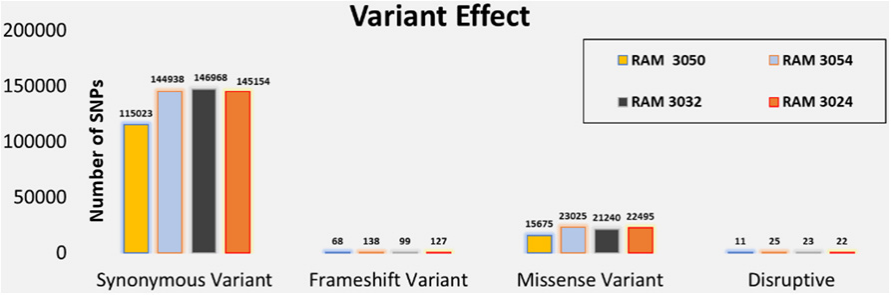
Snippy v4.6 was used to find single nucleotide polymorphisms in *E. marmotae*, using *E. coli* K-12 and *E. coli* J86-ST05 as a reference genome for WGS alignment. The functional effect of SNPs was investigated by SNPEff v4. 3 T, via Snippy.

Given that the genomes of *E. marmotae* and *E. coli* differ by about 10%, we may expect that functional differences may arise due to changes in expressed proteins resulting from single nucleotide polymorphisms (SNPs). Figure 2 shows the number and effect of SNPs in *E. marmotae* genomes in comparison to *E. coli* used as the reference genome. While most polymorphisms are synonymous and do not cause a change in expressed amino acids, approximately 15% of the sequence variants in *E. marmotae* caused frame-shift or non-synonymous sequence differences that may lead to functional change. Snippy highlights the specific genes with synonymous variations, missense mutations, frameshift mutations, disruptive mutations, and other genomic alterations.

To understand the potential biological impact of these genetic changes, we performed a functional gene ontology analysis on the genes containing these SNPs. SNP frequencies were estimated in 4 *E. marmotae* genomes compared to 3 *E. coli* strains to study the potential impact on gene function (Figure 2). The PANTHER GO slim classification categorized the genes based on their roles in biological processes, molecular functions, and cellular components. Metabolic and cellular processes seem especially prominent in these differences (Supplementary Figure 4A) and include catalytic activities (largest number of changes, in Supplementary Figure 4B). The molecular functions include genes involved in motility, chemotaxis, and biofilm formation, possibly suggesting changes in these functions. SNP analysis of these motility genes reveals that all examined loci harbor missense mutations. The functional categories identified by Panther GO-Slim Molecular Function analysis include bacterial flagellum-dependent motility (GO:0071973), chemotaxis (GO:0006935), and positive chemotaxis (GO:0050918), underscoring their roles in cellular movement and environmental response. All other functions are shown in Supplementary Table 5.

### 3.4 Serotype genes and virulence genes

**FIGURE 3:**
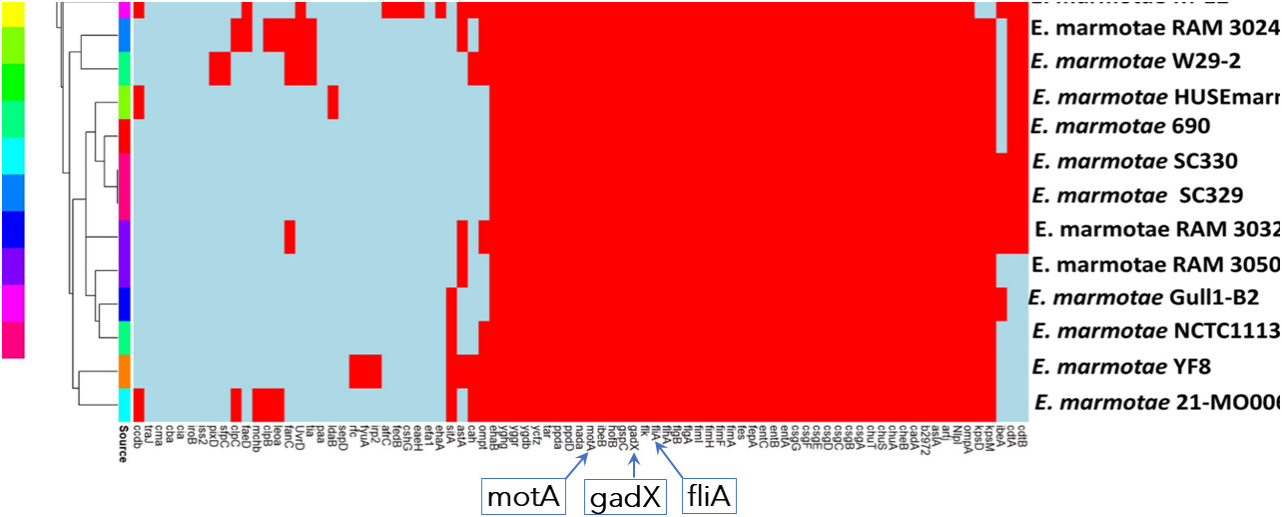
Virulence genes found in 18 *E. marmotae* genomes from Ram lab and GenBank using Virulence Finder and *E. coli* VF. The red color shows presence of the gene and the light blue color shows absence of the gene. The legend at the left side shows the sources of the *E. marmotae* isolates. Specific “boxed” genes whose names are enlarged at the bottom are the focus of analysis and discussion in the main text.

Serotype Finder indicates that these *E. marmotae* strains all had genes for the *fliC*-H56 flagellar antigen and varied in their O antigens, *fimH* type, and sequence types. Analysis for virulence genes in *E. marmotae* with VirulenceFinder and *E. coli* VF revealed a large number of putative virulence genes in *E. marmotae*, as illustrated in Figure 3. *E. marmotae* harbors numerous virulence genes, indicated by the fine print list across the bottom of the figure (a legible list with the number of genomes out of 18 is provided in Supplementary Table 6, including additional related genes not shown in Figure 3). In Figure 3, we have highlighted three genes*, fliA*, *motA*, and *gadX*, which were investigated further in subsequent bioinformatics and experimental analysis. The full list in Supplementary Table 6 includes genes involved in adhesion (*csg*A/B/D/E/F/G; *fimA/B/C/D/E/F/G/H/I*, *efa1*), nutritional/metabolic functions (e.g., iron acquisition, *entA/B/C/D/E/F/S*, *fepA/B/C/D/G* and *fes*), effector delivery systems (secretion systems type II, genes *gspC/D/E/F/G/H/I/J/K/L/M*; type III, T3SS; and type VI, T6SS), invasion (*kpsD*, *kpsM, ibeA, ibeB and ibeC*), toxins (*astA*, *cdt*, and *cdtA/B*), transport (*gadX*) and motility (e.g., *fliA*, *motA*, and others not shown). Some of these genes achieve their function directly; others achieve their functional role by being transcriptional regulators (e.g., *gadX* and *fliA*) and have also been characterized as antimicrobial resistance (AMR) genes, as described below. However, AMR genes are best found with AMR-dedicated software and databases.

### 3.5 Antibiotic resistance genes and sensitivity

#### 3.5.1 Antibiotic resistance genes

**FIGURE 4:**
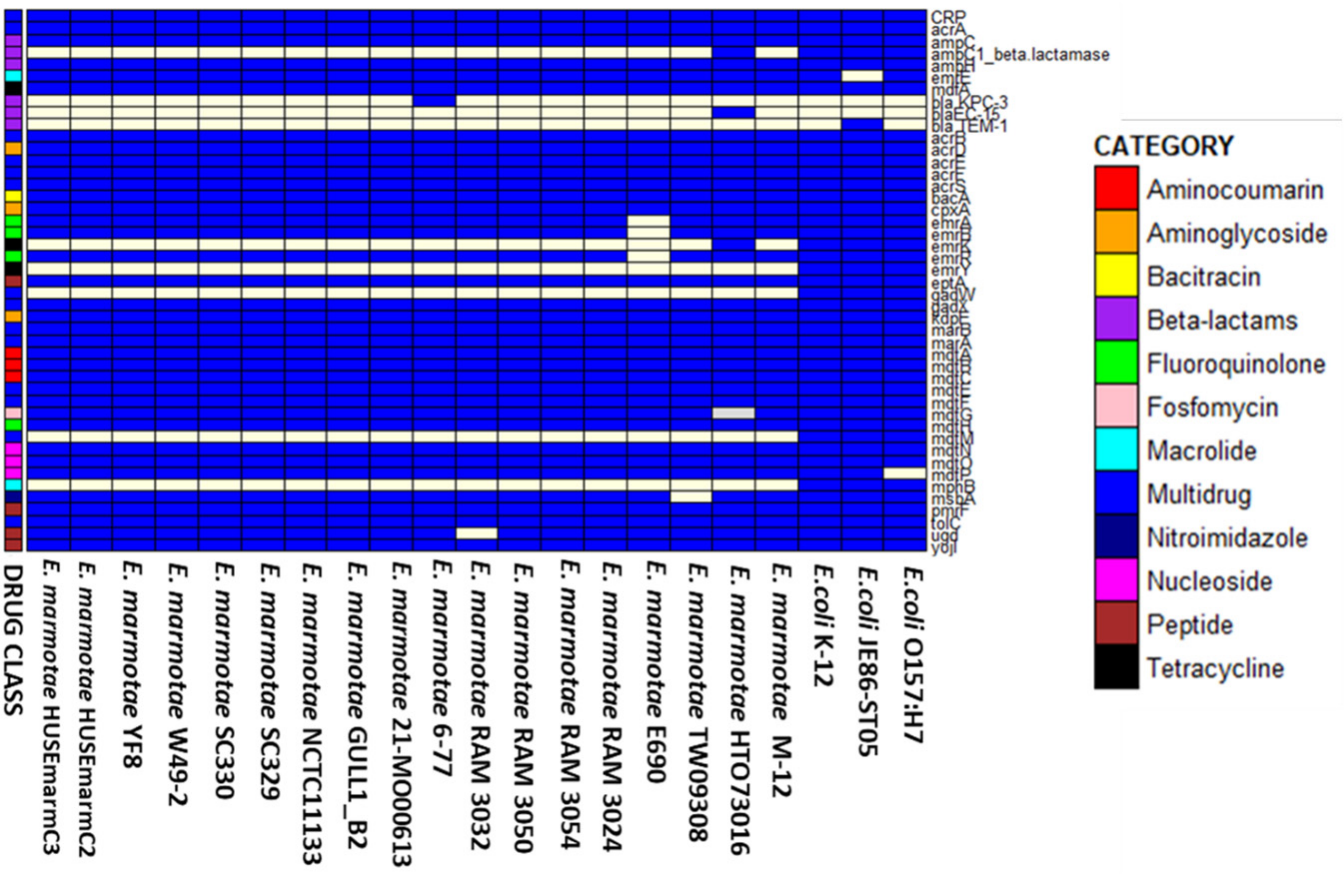
The presence of antibiotic resistance genes in 18 strains of *E. marmotae* and 3 strains of *E. coli*. The blue color shows presence of the gene, and white indicates the gene is absent. The legend at the left side indicates the drug category, according to the key at the right.

Figure 4 shows the AMR genes in the 18 *E. marmotae* genomes and in three *E. coli* genomes, with a minimal sequence identity to the CARD database of at least 80%. The AMR genes in *E. marmotae* exhibit 81–97% similarity to AMR genes in the CARD database, while *E. coli* strains show 94–100% similarity to the CARD database. These AMR genes include *ampC* and *ampH*, which confer resistance to cephalosporin-class and penicillin-like antibiotics (Jacoby, 2009). Some genes present in *E. marmotae* mediate multi-drug resistance, such as, *acrA, acrB, acrE, acrF, gadX, marA, mdtE, mdtF*, and *tolC*. Resistance to aminoglycosides includes *acrD, cpxA, kdpE*. Others include CRP, *mdtH, emrA, emrB, emrR, yojl, ugd, pmrf, eptA, bacA, emrE*, and *mdfA.* In this analysis, no AMR gene was present in *E. marmotae* that was not also present in *E. coli.* However, four AMR genes appear to be absent from all *E. marmotae* genomes examined but appear to be common in *E. coli* genomes. These genes (shown in Figure 4 as “all white” lines in the left part of the figure, and “all blue” in the three *E. coli* strains at the right) are *emrY*, *gadW*, *mdtM*, and *mphB*. Actually, *gadW* is present according to the pan-genome analysis; however, it is only 75% identical to the CARD database reference sequence and therefore below the minimal sequence identity used in the CARD comparison.

The presence of an AMR gene does not necessarily mean that they produce resistance, as some genes need to be expressed to achieve their function, while others may have diverged so much from their canonical sequence that they are unable to mediate antibiotic resistance. Given the relatively low percentage matches to the CARD database, compared to *E. coli*, we subjected these *E. marmotae* strains to tests of antibiotic sensitivity to a range of antibiotics to determine if these genes mediate antibiotic resistance in these isolates.

#### 3.5.2 Antibiotic sensitivity

**FIGURE 5:**
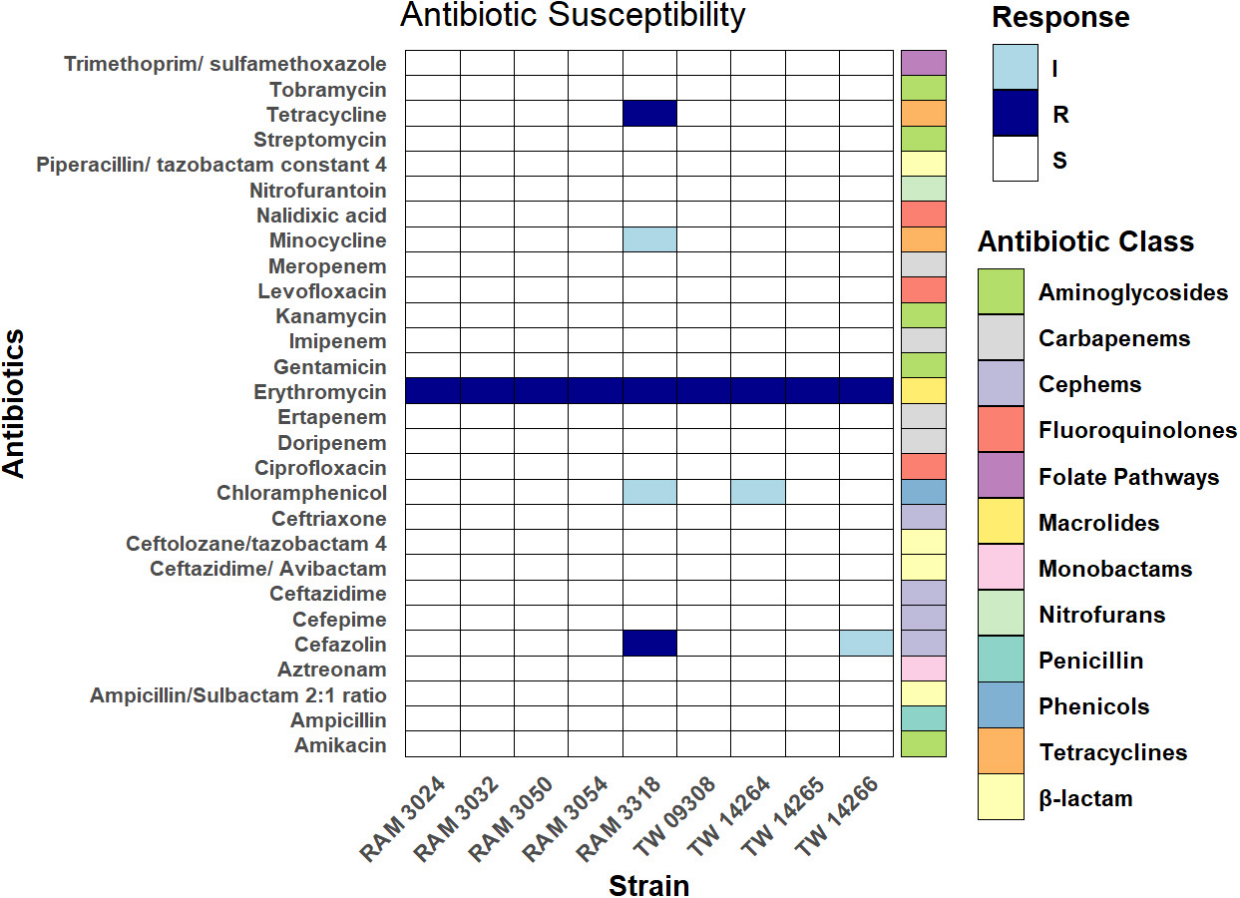
Antimicrobial susceptibility of *E. marmotae* strains to various antibiotics, tested on the Sensititre Antimicrobial Susceptibility System (24 antibiotics) and erythromycin, streptomycin, kanamycin and nalidixic acid tested by a broth dilution method. The dark blue, light blue, and white represent resistant, susceptible at mid-range concentrations, and susceptible, respectively.

Figure 5 presents the final susceptibility interpretations from both the Sensititre panel and the broth-dilution assays. On the Sensititre Antimicrobial Susceptibility System, most *E. marmotae* strains were susceptible to the majority of antibiotics. RAM 3318 exhibits resistance to tetracycline and cefazolin (Figure 5). Tests of the sensitivity of *E. marmotae* to other antibiotics using the broth dilution method revealed that both *E. marmotae* and *E. coli* are sensitive to nalidixic acid, kanamycin and streptomycin and resistant to erythromycin (Figure 5).

### 3.6 Motility

#### 3.6.1 Motility assays

**FIGURE 6:**
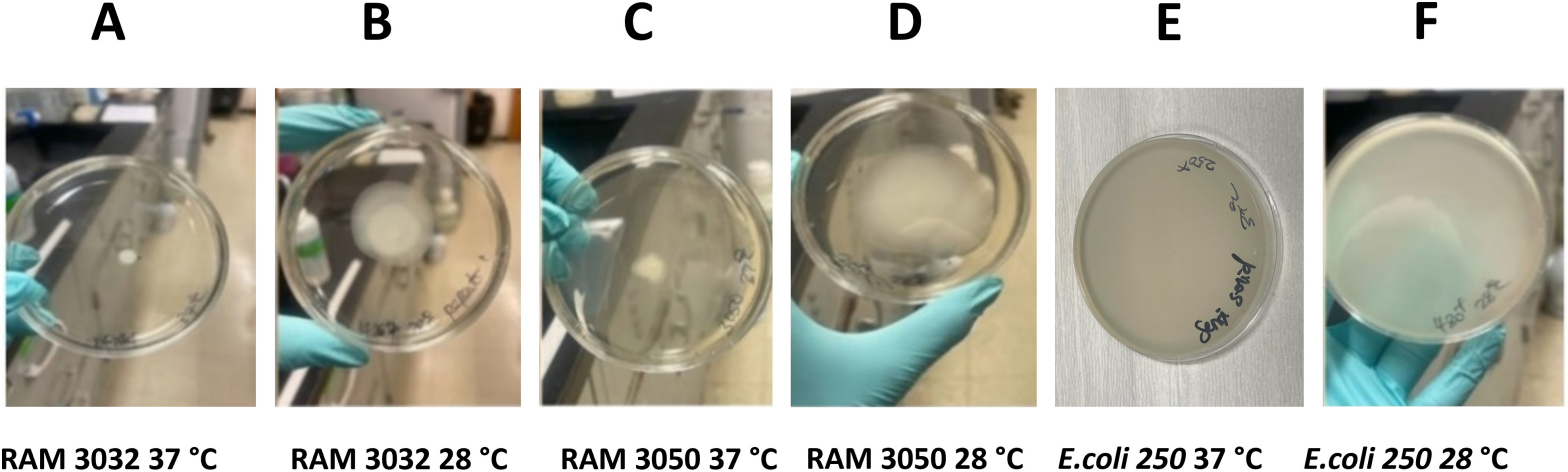
Representative examples of swim zone (spread) diameters of *E. marmotae* and *E. coli* after incubation for 24 h at different temperatures. **(A,C)** Swim zones of *E. marmotae* strain after incubation at 37°C. **(B,D)** Swim zones of *E. marmotae* after incubation at 28°C. Swimming zone diameters of *E. coli* strain diffuse over the entire plate both at **(E)** 37°C and **(F)** 28°C (the representative example shown here was incubated at 28°C).

**FIGURE 7:**
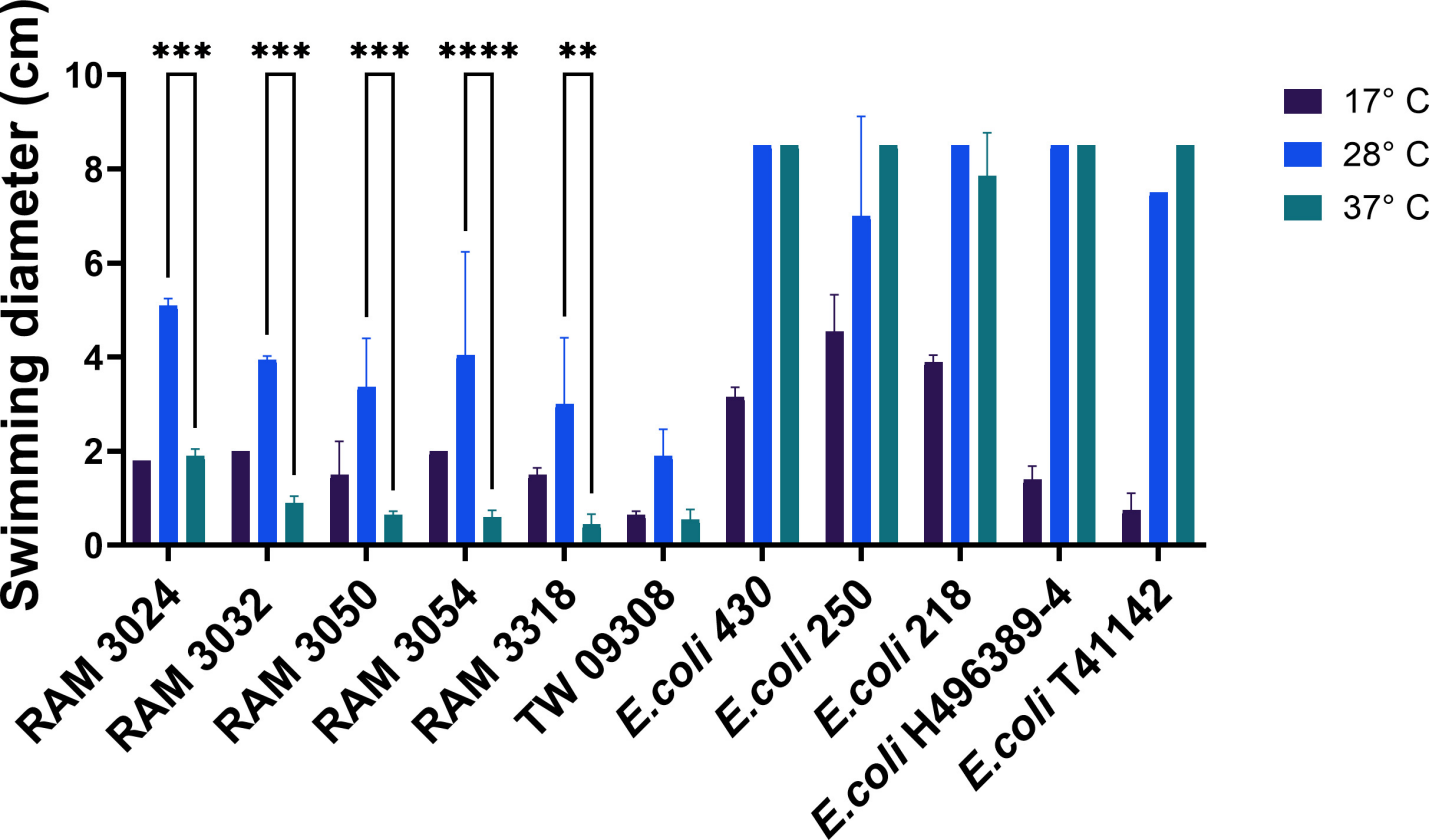
Motility determined by swim zone (spread) diameters of *E. marmotae* and *E. coli* after 24 h at 17, 28, and 37°C. The vertical bars represent the standard deviation. For *E. marmotae*, motility at 28°C is significantly greater than at 37°C (**P < 0.01, ***P < 0.001; ****P < 0.0001; two way ANOVA, followed by Tukey multiple comparison tests). Statistical analysis comparing *E. coli* and *E. marmotae* at 28°C and 37°C using the Kruskal–Wallis test showed a significant difference between the two species (*P* < 0.001). For clarity, the statistical significance of the 17°C result is not marked on the illustration but was in every case significantly different from 28°C for both *E. marmotae* and *E. coli.*

To address the potential roles of motility in pathogenicity and in adaptations of *E. marmotae* to infect mammalian hosts, we investigated *E. marmotae* motility at various temperatures (17, 28, and 37°C) and compared it to *E. coli*, as illustrated by the representative examples in Figure 6. *E. marmotae* had a smaller spread diameter at 37°C (Figures 6A,C), indicating lower motility, in comparison to *E. coli* (Figure 6E). The motility of *E. marmotae* increased at 28°C, in comparison to 37°C (Figures 6B,D), but was still significantly less than *E. coli*, whose motility was not significantly reduced at 28°C, as summarized graphically in Figure 7. The motilities of both species were greatly reduced at 17°C. The pilot studies on time course assay in Supplementary Figure 5A, shows reduced migration at 37°C from the inoculation point over 24 h. Motility tests performed on clinical strains of *E. marmotae* isolated from humans (Sivertsen et al., 2022) revealed a similar pattern of reduced motility at 37°C consistent with the findings in environmental strains (Supplementary Figure 5B).

#### 3.6.2 Bacteria growth analysis

Since the larger spread diameter of *E. marmotae* at 28°C could potentially be due to differences in growth at various temperatures, bacterial growth rates were assessed by growth curve analysis at different temperatures. For *E. marmotae*, OD_600_ increased more rapidly at 37°C than at 28°C until the stationary phase was reached, indicating faster growth at the host-relevant temperature (Supplementary Figures 6A–E). This is opposite to what might be expected if the larger spread diameter observed at 28°C was due to greater growth at that temperature. Instead, it suggests that the increased spread diameter at 28°C is likely motility-driven rather than growth-driven.

We also compared the growth of all the tested *E. marmotae* strains with that of the *E. coli* strains over 24 h. At all-time points, the growth of *E. coli* was greater (Supplementary Figure 6F).

#### 3.6.3 Expression levels of flagellar genes

**FIGURE 8:**
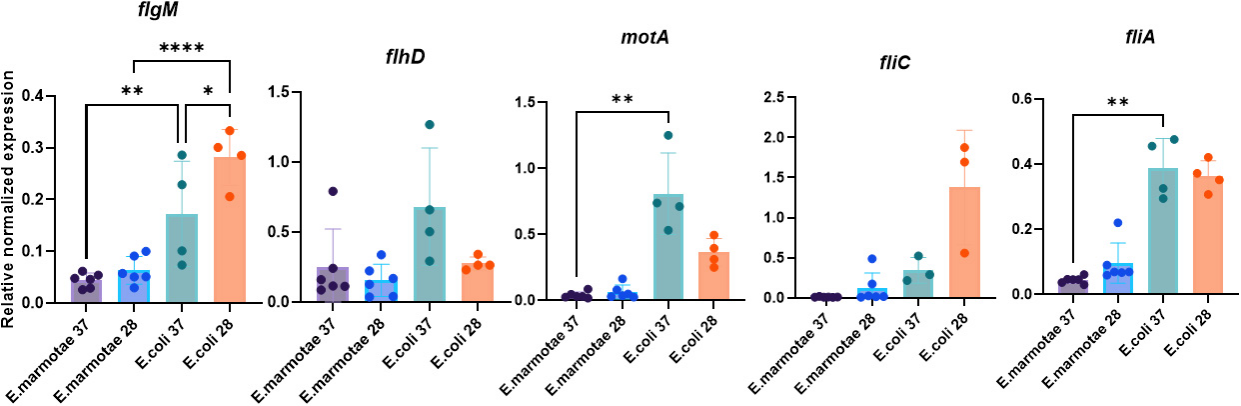
Interspecies comparison of gene expression in *E. marmotae* (*n* = 6 strains) and *E. coli* (*n* = 4 strains) at 37 and 28°C. Expression of each of the five genes is illustrated, normalized in comparison to *adk*. In general, the expression of each gene is higher in *E. coli* than in *E. marmotae* at both temperatures (as affirmed when the expression of all five genes is considered together; see text); however, for each gene, differences are statistically significant only where indicated by asterisks. Bars represent means with SD. Asterisks represent *p-*values as follows: *P = < 0.05, **P = 0.005, ****P = < 0.0001, determined by Kruskal-Wallis test.

To test the hypothesis that reduced motility of *E. marmotae* compared to *E. coli* may be caused by reduced expression of genes involved in motility, we analyzed the expression of *motA*, *fliC, flgM, flhD*, and *fliA*, in *E. marmotae* and *E. coli* incubated at both 28 and 37°C using RT-qPCR, normalized relative to the expression of the housekeeping gene *adk*. The normalized median level of expression of the five motility genes in *E. marmotae* was 0.096 at 28°C in *E. marmotae*, significantly lower than 0.363 at 28°C in *E. coli* (*p* = 0.0079, Mann-Whitney test). At 37°C, the median expression of these genes was 0.044 in *E. marmotae* and 0.386 in *E. coli* at 37°C (*p* = 0.016, significant with reference to the Bonferroni-corrected p of 0.025). The overall lower expression of these genes in *E. marmotae* is consistent with the hypothesis that the lower motility of *E. marmotae* is due to lower gene expression for these genes than in *E. coli*. This lower expression in *E. marmotae* than in *E. coli* is true for all of the genes considered individually as well, as summarized for all strains in Figure 8 and for each strain graphed separately in Supplementary Figure 7.

An interesting specific strain of *E. marmotae* was strain RAM3024, which showed the largest swimming diameter of any *E. marmotae* strain tested (Figure 7). This strain also showed a higher expression of motility genes in comparison to other *E. marmotae* strains For the five genes examined here the relative normalized expression at 28°C was *motA*, 0.164; *fliC*, 0.488; *flgM*, 0.100; *flhD*, 0.338; and fliA, 0.220; while expression at 37°C was *motA*, 0.085; *fliC*, 0.021; *flgM*, 0.062; *flhD*, 0.891; and fliA, 0.061 (see also Supplementary Figure 7). Each value is the average of 5 replicate extractions and RT-qPCR measurements.

**FIGURE 9:**
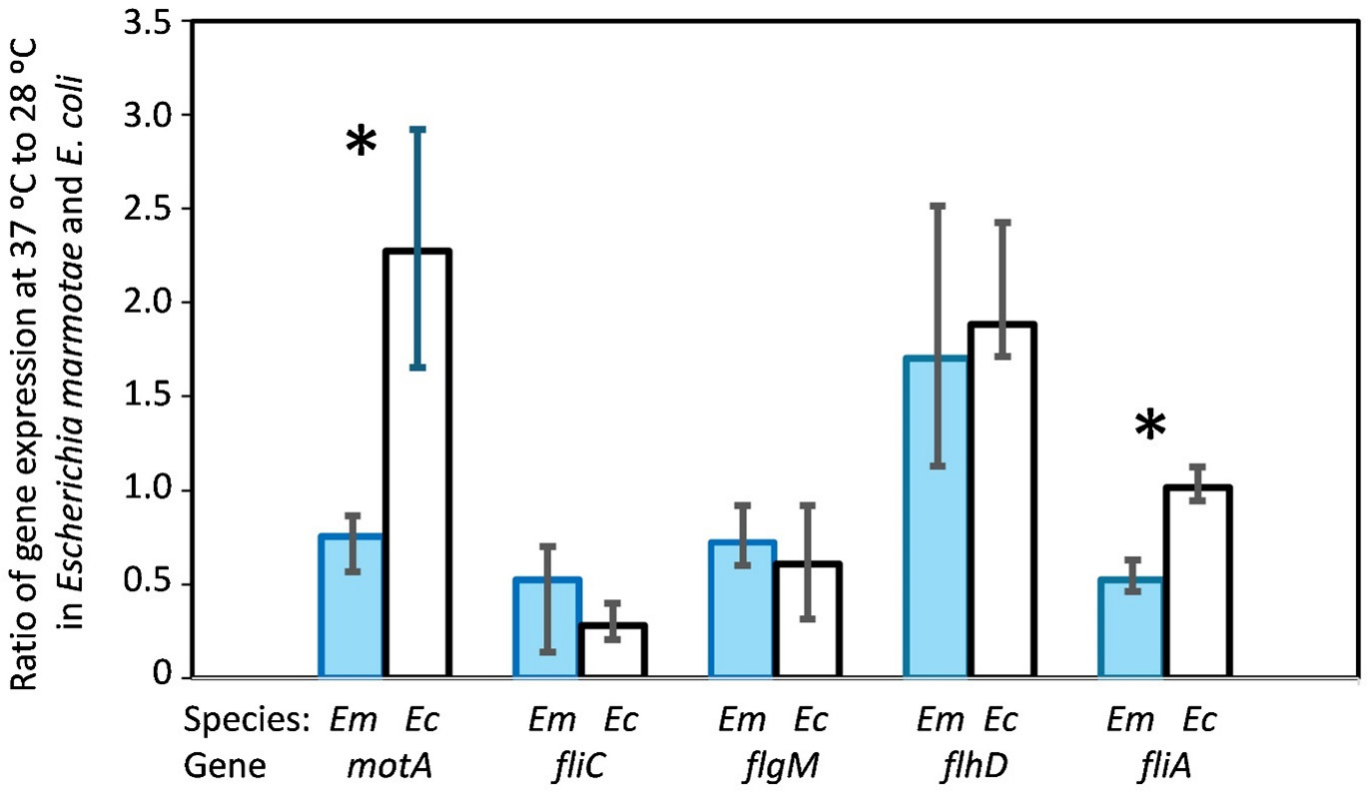
Effect of temperature on gene expression, as determined by the ratio of expression (37°C:28°C) for each of five genes involved in motility, compared between *E. marmotae* and *E. coli*. The ratio was determined for each of 6 strains of *E. marmotae* and for each of 4 strains of *E. coli*. Bars represent medians and interquartile ranges, and * indicates *p* = 0.0095 on a Mann-Whitney test. The Bonferroni corrected *p* value for significance for the 5 Mann-Whitney tests for this analysis is *p* = 0.01.

Comparing the effects of temperature within each species on expression of these motility genes presents a more nuanced result that is more complicated to analyze because the “baseline” expression is at such different levels between *E. marmotae* and *E. coli*. Therefore, we compared the *ratio* of expression (37°C: 28°C) in each strain, as summarized in Figure 9. Higher temperature decreased the expression of *fliA* and *motA* in *E. marmotae* (median ratios of 0.5 and 0.8, respectively), compared to no change (*fliA*) or increased expression (*motA*) of these genes in *E. coli* at the higher temperature significantly higher ratios of *fliA* = 1.0 (*p* = 0.0095, Mann-Whitney test), and *motA* = 2.3 (*p* = 0.0095, Mann-Whitney test). *fliC* also consistently had lower expression at 37 °C than 28 °C in *E. marmotae* (median ratio = 0.5), but this was no different from the comparable temperature-dependent change in *E. coli* (median ratio = 0.3). Except for one strain of *E. coli*, *flgM* was also lower or unchanged at 37 °C compared to 28 °C in *E. marmotae* and *E. coli*, with median ratios of 0.7 and 0.6, respectively. In contrast, the effect of temperature on *flhD* expression was increased expression at 37 °C for both *E. marmotae* (median ratio of 37°C:28°C expression = 1.7) and *E. coli* (median ratio = 1.9). Except for *flhD*, the expression of motility genes in *E. marmotae* seem generally to decrease at 37 °C, compared to 28 °C, and for two of these genes, *fliA*, and *motA*, the temperature effect on gene expression is significantly different from *E. coli*.

### 3.7 Biofilm formation

**FIGURE 10:**
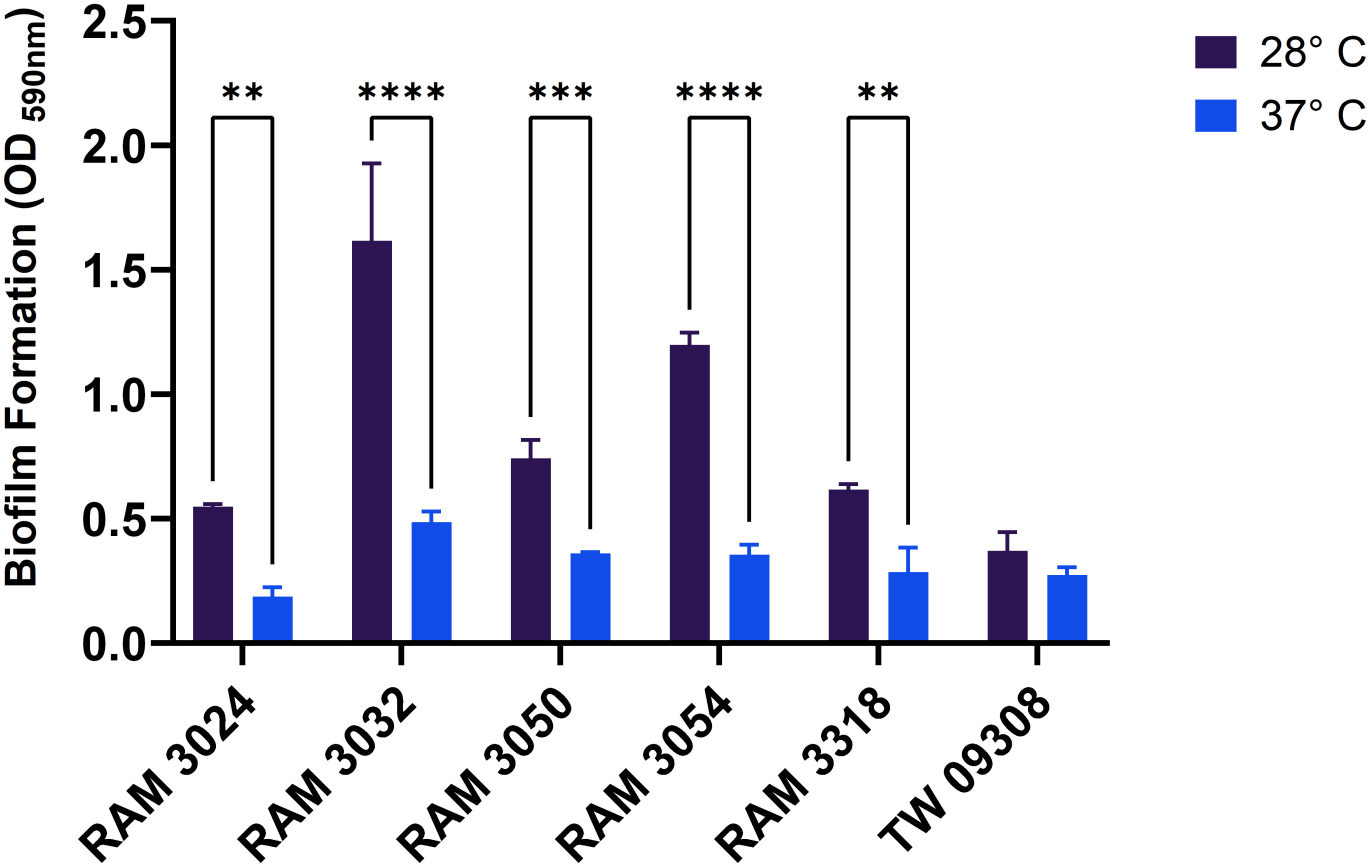
Biofilm formation of environmental *E. marmotae* strains in LB Broth for 48 h at 37°C and 28°C. The biofilm was stained with 0.1% crystal violet, extracted in 30% acetic acid, and the OD_590_ nm was measured. The bars and error bars represent the mean ± standard deviation. **P < 0.01, ***P < 0.001, ****P < 0.0001; two-way ANOVA followed by Sidak’s multiple comparison.

*E. marmotae* produced significantly more biofilm at 28°C (presence of ring formation at the air-liquid interface) compared to 37°C (little or no ring formation observed), as illustrated in Figure 10 and Supplementary Figure 8. All *E. marmotae* strains demonstrated this feature compared to the control medium (no bacteria strain present). Clinical strains of *E. marmotae* from Sivertsen et al. (2022) also revealed similar phenotypic traits of biofilm formation (Supplementary Figure 9).

## 4 Discussion

Among the *Escherichia* cryptic clades, *E. marmotae* is the most studied; however, much is still unknown. This paper adds five whole genome sequences to the approximately 50 in GenBank. Although previously mined for insights, our analysis adds new observations at the genomic level, particularly on the roles antibiotic resistance and motility genes may play in the pathogenic potential of *E. marmotae*. This study also reports new experimental observations on temperature-dependent changes in *E. marmotae* motility, motility gene expression, and biofilm formation that differentiate *E. marmotae* from *E. coli*.

### 4.1 Conserved and differential features of *E. marmotae* sequences

A challenge in the study of the prevalence of *E. marmotae* is their phenotypic similarity to *E. coli* on practically all standard tests. Practically every isolate of *E. marmotae* has been discovered by screening isolates identified by standard methods as *E. coli* and then subsequently discovering that sequences are 10% different (most genes) from *E. coli* or unique in their 16S rDNA sequences (Clermont et al., 2011; Sivertsen et al., 2022; Sinha et al., 2023). Despite early claims to growth of *E. marmotae* on nutrients that do not support *E. coli* (e.g., glycerol in Liu et al., 2015), those media proved not to be specific in our hands (unpublished data). The discovery of unique antibiotic resistance in *E. marmotae* would advance this goal considerably, so our initial foray into genome analysis (section 3.5) was to investigate the antibiotic resistome of *E. marmotae* and then to test antibiotic sensitivity with the Sensititre system. These results revealed (a) four AMR genes that occur in *E. coli* but are absent in *E. marmotae*, but (b) so far, no AMR genes that are present in *E marmotae* but absent in *E. coli*. A limitation of this ‘reference gene search’ is that the *E. marmotae* sequences vary on average about 10% from *E. coli* and could in some cases appear to be absent (even if present) because they have fallen below the threshold of 85% identity to the CARD database. Even for AMR genes that were > 85% identical to the CARD database, none of the potential resistances were reflected in phenotypic MIC testing, suggesting that further work is needed under conditions not covered by the Sensititre system.

One future direction may be pH sensitivity. The *gadX* gene, present in both the virulome (Figure 3) and AMR gene screening (Figure 4), is a transcriptional activator that works in concert with *gadW* to confer acid resistance on *Escherichia* strains, enabling them to resist the acidic environment of the vertebrate stomach (Tucker et al., 2003; Sheikh et al., 2021). Tucker et al. (2003) found that mutating *gadW* (unbeknownst to them, a possible mimic of *E. marmotae*) increased the resistance of *E. coli* to acidic environments. Sheikh et al. (2021) noted that *gadX*/*gadW* transcriptional control is important in EHEC *Escherichia* pathogenicity. Other genes lacking in *E. marmotae* in comparison to *E. coli* (Figure 4) include *emrY*, associated with the regulation of efflux pumps involved in surviving extreme-acid conditions (Deininger et al., 2011) and *mdtM*, a gene that may contribute to alkaline pH tolerance (Holdsworth and Law, 2013). In future experiments, therefore, the impacts of pH on *E. marmotae* should be tested.

*E. marmotae* lacked *mphB* (included in Figure 4 because of its presence in *E. coli*). *mphB* can mediate resistance to macrolides such as erythromycin. *E. marmotae* is nevertheless resistant to erythromycin, which may be mediated by other genes, such as *mphA* (Noguchi et al., 2000). Curiously, the type specimen for *E. marmotae*, strain HTO73016, appears to be atypical compared to other *E. marmotae* in Figure 4, having additional AMR gene markers (*ampC1*, *blaEC.15*, and *emtK*).

In fact, *E. marmotae* has been found in numerous mammals and birds, besides the marmot from which the type specimen was described. Our laboratory isolated it from raccoon feces (Ram et al., 2007) and several publications found it most common in birds (Blyton et al., 2015; Souguir et al., 2023; Binsker et al., 2024). Like *E. coli*, a human pathogen that is also a vertebrate commensal and often collected in the environment, *E. marmotae* can adapt to a human host environment. Accordingly, *E. marmotae* has also been found in clinical settings (Clermont et al., 2011; Lefort et al., 2011; Sivertsen et al., 2022; Sinha et al., 2023). So, a reasonable question to ask is what unique functional properties does *E. marmotae* have that might distinguish *E. marmotae* infections from those of *E. coli?*

### 4.2 Motility and motility gene expression responses to temperature

Flagellar-driven motility can contribute to the virulence of bacteria by facilitating their spread to new surfaces (Guttenplan and Kearns, 2013; Vilas Boas et al., 2024). However, as described further below, flagella can also elicit immune responses, so a loss of motility due to reduced expression of flagella may, in some cases, increase pathogenic potential (62). These contradictory implications of flagellar-driven motility on pathogenicity motivated us to study motility and the expression of genes associated with it. We found that *E. marmotae* is less motile than *E. coli*, and the motility of *E marmotae* is affected differently by temperature than in *E. coli*. We hypothesize that *E. marmotae* might therefore produce a different disease profile than *E. coli* when infections do occur.

**FIGURE 11:**
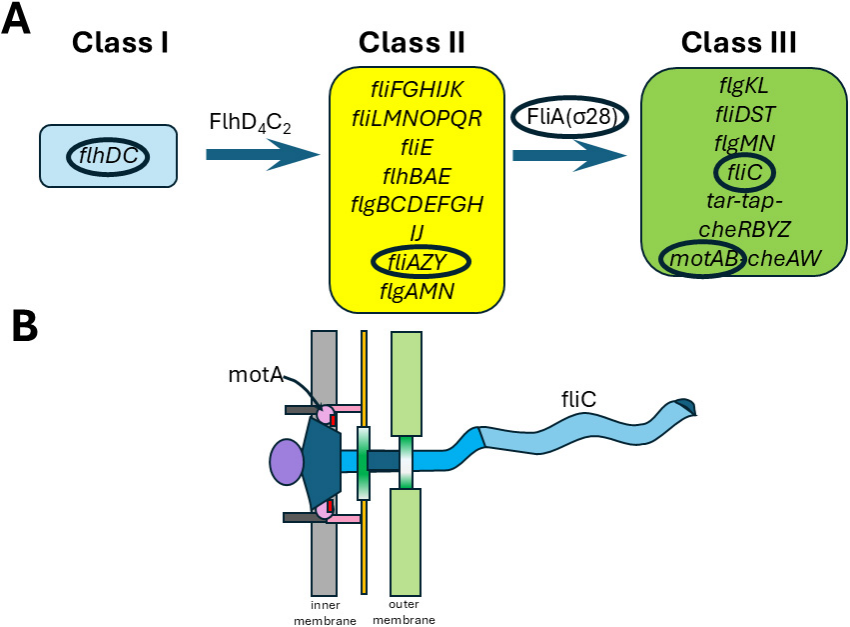
Flagellar gene classes and structural components. **(A)** The genetically defined hierarchy of flagellar genes. **(B)** Major components of the flagella and the location of the flagellar gene products. Genes whose expression were studied in this paper are circled.

To investigate whether expression of motility genes might underlie differences in motility between *E. marmotae* and *E. coli*, we examined the expression of genes involved in motility, selected from the three classes of flagellar genes (Figure 11): Class I (e.g., *flhD)*, Class II *(fliA, flgM)*, and Class III *(fliC, motA*). Motility among *Escherichia* has been extensively characterized across various non-pathogenic and pathogenic strains (Sudarshan et al., 2021), including *E. coli* MG1655 (Gómez-Gómez et al., 2007) and avian pathogenic *E. coli* (APEC) strains such as IMT5155 (Aleksandrowicz et al., 2024). Among clinical isolates, motility is mostly present, except in extensively drug-resistant (XDR) strains, which often show impaired motility (El-baz et al., 2022). Deletion of *fliA* or *motA* independently leads to loss of motility and absence of flagella (Lane et al., 2005; Vilas Boas et al., 2024). Consistent with these findings, transmission electron microscopy (TEM) of *E. marmotae* revealed the presence of flagella at 28°C (Supplementary Figure 10) but not at 37°C (Binsker et al., 2024).

Temperature affects motility differently in *E. marmotae* than in *E. coli*. Many virulence-associated genes in pathogens are regulated by temperature, with changes occurring at the host-relevant temperature (Shapiro and Cowen, 2012; Lam et al., 2014). However, higher temperature effects on motility gene expression varies, with some genes decreasing (e.g., *fliC* and *flgM* had 37°C:28°C ratios below 1.0 in Figure 9), while others increase (e.g., in *E. coli*, *flhD* and *motA*; 37°C:28°C ratios > 1.0 in Figure 9). The ratios of expression at the two temperatures are calculated for each strain in Figure 9 before averaging, which is a more sensitive measure of the effect of temperature than averaging the expression across several strains and then taking the average, as in Figure 8. Both methods show the same direction of change for each gene. For example, the mean of motA expression in *E. coli* averages about twice as high at 37°C as at 28°C in both Figures 8, 9, and the ratio calculation in Figure 9 shows a significant difference of *E. coli* from *E. marmotae* that is not present with the less sensitive statistical method used in Figure 8. *E. marmotae* not only has lower overall motility compared to *E. coli* but its motility also decreases at 37°C, which may explain why previous publications were contradictory about whether *E. marmotae* was motile (Zhurilov et al., 2023) or non-motile (Liu et al., 2015). *E. marmotae* may switch between motile states, triggered by temperature changes when it enters a host. A limitation of this study is that we did not measure the expression of numerous other genes involved in motility (many are listed in the second paragraph of section 3.2), which may play greater roles in temperature dependent changes in motility that we observed.

Some pathogenic bacteria stop producing flagella at host temperatures in order to avoid host immune responses. *Listeria monocytogenes* is motile below 30°C and loses flagella motility at 37°C (Kathariou et al., 1995; Abdulkadieva et al., 2023). Similarly, *Yersinia enterocolitica* is less motile at 37°C than at 25°C (Kapatral and Minnich, 1995). Even *E. coli* decreases its motility and expression of specific genes at somewhat higher temperatures (e.g., 42°C, Rudenko et al., 2019) than we studied in *E. marmotae*. Rudenko et al. (2019) suggested that the decrease in expression of flagellar structural genes at 42°C might reduce their exposure to immune responses. *Salmonella* Paratyphi A also reduces motility and flagellar gene expression at 42°C (Elhadad et al., 2015), which may also reduce immune responses (Yue et al., 2023). While this putative protection in *E. coli* and *Salmonella* occurs at febrile temperatures (42°C) associated with active immune responses, *E. marmotae* might anticipate this immune response by reducing its expression of structural genes such as *motA* and the sigma factor *fliA* (regulator of Class III genes) at 37°C.

Rudenko et al., 2019 observed an insignificant rise in mRNA expression for *fliA* between 27 and 37°C and then a significant decrease in *fliA* mRNA expression at 42°C in *E. coli*. We also observed no significant change in *E. coli fliA* mRNA between 28 and 37°C, but in *E. marmotae fliA* mRNA decreased at 37°C in comparison to 28°C, a significantly different response than we measured in *E. coli* (Figure 9). A decrease in *fliA*, which regulates Class III gene expression, would be expected to be accompanied by decreases in expression of genes such as *motA*. In our studies, *motA* expression trended downward at 37°C in *E. marmotae*, significantly less than the two-fold increase observed in *E. coli* (Figure 9). Rudenko et al. (2019) also observed an upward trend in *motA* expression between 27 and 37°C, but then a significant 12-fold decrease in *motA* expression at 42°C. Quantitative differences of our *E. coli* results from previous publications might be due to the fact that our *E. coli* are environmental isolates, which might differ from the laboratory strains studied by others. The quantitative differences, notwithstanding, the downstream regulation of *motA* expression, possibly related to *fliA* expression, seems to be common in motility regulation. *E. marmotae* may thereby avoid immune responses by decreasing motility gene expression.

Given its low motility at 37°C, *E. marmotae* biofilm formation may be especially important in its invasion. While motility is often critical for bacterial dissemination, in later stages of infection, biofilm formation plays a vital role in persistence and immune evasion (Chaban et al., 2015; Sahoo and Meshram, 2024). For *E. coli*, biofilms have been invoked as important in urinary tract infections (Buck et al., 2021). Despite decreases at 37°C, every strain of *E. marmotae* that we tested formed weak biofilms at 37°C. This is consistent with previous findings that *E. coli* cryptic relatives formed stronger biofilms at a lower temperature (Ingle et al., 2011). Some *E. coli* are very good biofilm formers at low temperatures (Nesse et al., 2014; Mathlouthi et al., 2018). The motility gene *FliA* may also play a role in the formation of biofilms, as its overexpression in *E. coli* can mediate increases in biofilm formation (Buck et al., 2021). Future directions will investigate the genes involved in temperature regulation of motility and biofilm formation and how they may affect infection.

Overall, these findings emphasize the complexity of *E. marmotae* and its potential to thrive in diverse environments. Our work expands the understanding of *E. marmotae*, especially its temperature-dependent behavior. Whether these functional differences result in infections that differ from *E. coli* remains to be determined.

## Data Availability

The datasets presented in this study can be found in online repositories. The names of the repository/repositories and accession number(s) can be found in the article/Supplementary material.

## Footnotes

1
https://jspecies.ribohost.com/jspeciesws/

2
https://www.mgc.ac.cn/VFs/

3
https://www.genomicepidemiology.org/services/
