# Supplementary material for: Genome organization, virulence genes, and temperature-dependent motility of an emerging pathogen, Escherichia marmotae
Source: Front Microbiol. 2025 Dec 17;16:1729604. doi: 10.3389/fmicb.2025.1729604 (PMC12754014; doi:10.3389/fmicb.2025.1729604)
Supplement: Supplementary file 1 [file Data_Sheet_1.docx]

**Title**: Genome Organization, Virulence Genes and Temperature-Dependent Physiology of an Emerging Pathogen, *Escherichia marmotae*

Authors: Pelumi M. Oladipo*^1,2^, Ali M. Jomaa^1^, Jeffrey H. Withey^2^, Jeffrey L. Ram^1^

^1^Department of Physiology, Wayne State University School of Medicine

^2^Department of Biochemistry, Microbiology, and Immunology, Wayne State University School of Medicine

*corresponding author: Pelumi M. Oladipo, pelumioladipo@wayne.edu

**Table S1: Assembly metrics of WGS of *E. marmotae* strains**

| **Strains^a^** | **Contigs** | **Total Length (bp)** | **GC (%)** | **N50 (bp)^b^** | **N75 (bp)^b^** | **L50^b^** | **L75^b^** |
| --- | --- | --- | --- | --- | --- | --- | --- |
| RAM 3024 | 151 | 4902904 | 50.4 | 199096 | 152690 | 7 | 14 |
| TW 14264 | 83 | 4507182 | 50.4 | 330820 | 199725 | 5 | 10 |
| RAM 3032 | 97 | 4504983 | 50.39 | 235606 | 159259 | 6 | 12 |
| RAM 3050 | 112 | 4388739 | 50.46 | 205833 | 120567 | 8 | 14 |
| RAM 3054 | 408 | 5173310 | 50.26 | 209045 | 117349 | 9 | 17 |
| RAM 3318 | 74 | 4614133 | 50.37 | 177536 | 96904 | 8 | 24 |

^a^RAM #### is the original strain designation, but after regrowth and distribution by the Michigan State University archive the equivalent strains are as follows: RAM 3318 = TW14267, RAM 3024 = TW 14263, RAM 3032 = TW 14264, RAM 3050 =TW 14265, and RAM 3054 =TW 14266.

^b^The meaning of column headings are defined by Quality Assessment Tool (QUAST) (62) in which N50 and N75 are the length for which the collection of all contigs of that length or longer covers at least 50% or 75%, respectively, of the assembly; L50 and L75 are the number of contigs equal to or longer than N50 and N75, respectively. In other words, L50 is the minimal number of contigs that cover half the assembly.

**Table S2: The WGS details of *E. marmotae* and *E.coli* used in Bioinformatics analysis**

| **s/n** | **Strains** | **GenBank Accessions** | **Size** | **CDS^a^** | **Collec-tion Year** | **Isolation Country** | **Host Common Name** |
| --- | --- | --- | --- | --- | --- | --- | --- |
| 1 | *Escherichia marmotae* 21-MO00613 | JAHCSB000000000 | 5223662 | 5426 | 2020 | Germany | Pig |
| 2 | *Escherichia marmotae* M-12 | JAPMJP000000000 | 4903676 | 5043 | 2021 | Russia | Vole |
| 3 | *Escherichia marmotae* Gull1_B2 | JALXJT000000000 | 4758314 | 4779 | 2003 | USA | Ring-Billed  Gull |
| 4 | *Escherichia marmotae* strain E690 | JABXGM000000000 | 4303797 | 4146 | 2019 | Spain | Cow |
| 5 | *Escherichia marmotae* strain SC330 | QOOC00000000 | 4574552 | 4500 | 2005 | USA | Plumbing |
| 6 | Escherichia marmotae strain SC329 | QOOD00000000 | 4594109 | 4564 | 2005 | USA | Plumbing |
| 7 | *Escherichia marmotae* HUSEmarmC2 | CAKAEI000000000 | 4543923 | 4436 | 2021 | Norway | Human |
| 8 | *Escherichia marmotae* HUSEmarmC3 | CAKAEJ000000000 | 4657376 | 4616 | 2021 | Norway | Human |
| 9 | *Escherichia marmotae* YF8 | CP072689,  CP072690 | 4669757 | 4520 | 2001 | Australia | Dog |
| 10 | *Escherichia marmotae* strain 6-77 | JAAFFT000000000 | 4818974 | 4852 | NA | Portugal | Human |
| 11 | *Escherichia marmotae* W49-2 | CP093239,  CP093240,  CP093241 | 5200167 | 5300 | 2021 | Poland | Not identified |
| 12 | *Escherichia marmotae* HT073016 | JNBP00000000 | 4617373 | 4506 | 2012 | China | Plumbing |
| 13 | *Escherichia marmotae* strain NCTC11133 | LR134340.1 | 4450344 | 4067 | ND | USA | NA |
| 14 | *Escherichia marmotae* TW 09308 | NZ_AEME01000001 | 4809826 | 4633 | ND | USA | Freshwater  beach |
| 15 | *Escherichia marmotae* RAM 3032 | JBNVMU000000000^b^ | 4504983 | 4100 | 2005 | USA | Sheridan Rd, Storm Sewer |
| 16 | *Escherichia marmotae* RAM 3050 | JBNVMV000000000^b^ | 4388739 | 3991 | 2005 | USA | Buckingham  Rd Storm  Sewer |
| 17 | *Escherichia marmotae* RAM 3054 | JBNVMW000000000^b^ | 5173310 | 4822 | 2005 | USA | Buckingham  Rd Storm  Sewer |
| 18 | *Escherichia marmotae* RAM 3024 | JBNVMX000000000^b^ | 4902904 | 4583 | 2005 | USA | Raccoon |
| 19 | *Escherichia coli* str. K-12 substr. MG1655 | CP009685 | 4636831 | 4651 | 2014 | USA | NA |
| 20 | *Escherichia coli* O157:H7 str. 2011EL-1107 | JHLK00000000 | 5414139 | 5479 | 2011 | USA | NA |
| 21 | *Escherichia coli* O86 strain JE86-ST05 | BHVN01000001 | 5413008 | 5567 | 2015 | Asia | Human |

^a^CDS is a sequence of nucleotides that corresponds with the sequence of amino acids in a protein (63).

^b^The RamLab strains, while not identified with the strain numbers in the original paper (Ram et al. 2007) were, in fact, from the indicated sources and archived with these strain numbers.

The Accession number of RAM 3318 but not used in bioinformatics analysis is JBNVMY000000000.

Table S3: List of other *E.coli* strains used in this study

| S/N | Strains | Source |
| --- | --- | --- |
| 1 | *Escherichia coli* 250 | Environment |
| 2 | *Escherichia coli* 430 | Environment |
| 3 | *Escherichia coli* 218 | Environment |
| 4 | *Escherichia coli* H496389-4 | Human |
| 5 | *Escherichia coli* T41142 | Human |

Table S4: Whole Genome Percentage Identities Between *E. marmotae* and *E. coli* Strains. See supplemental Excel file “Table Suppl 3.xlsx”

Table S5: Pangenome of 18 strains of *Escherichia marmotae*. See supplemental Excel file “Supp 4 pangenome *E. marmotae*.xlsx”

Table S6: PANTHER GO-Slim Function of Non-Synonymous Variation in *E. marmotae.* See supplemental Excel “Table S5-Panther Go Slim Function”

Table S7: Putative Virulence Factor of *E. marmotae*. See supplemental Excel “Virulence factor *E. marmotae”* (a legible list with the number of genomes out of 18 is provided in Table S6, including additional related genes not shown in Figure 4)”

**
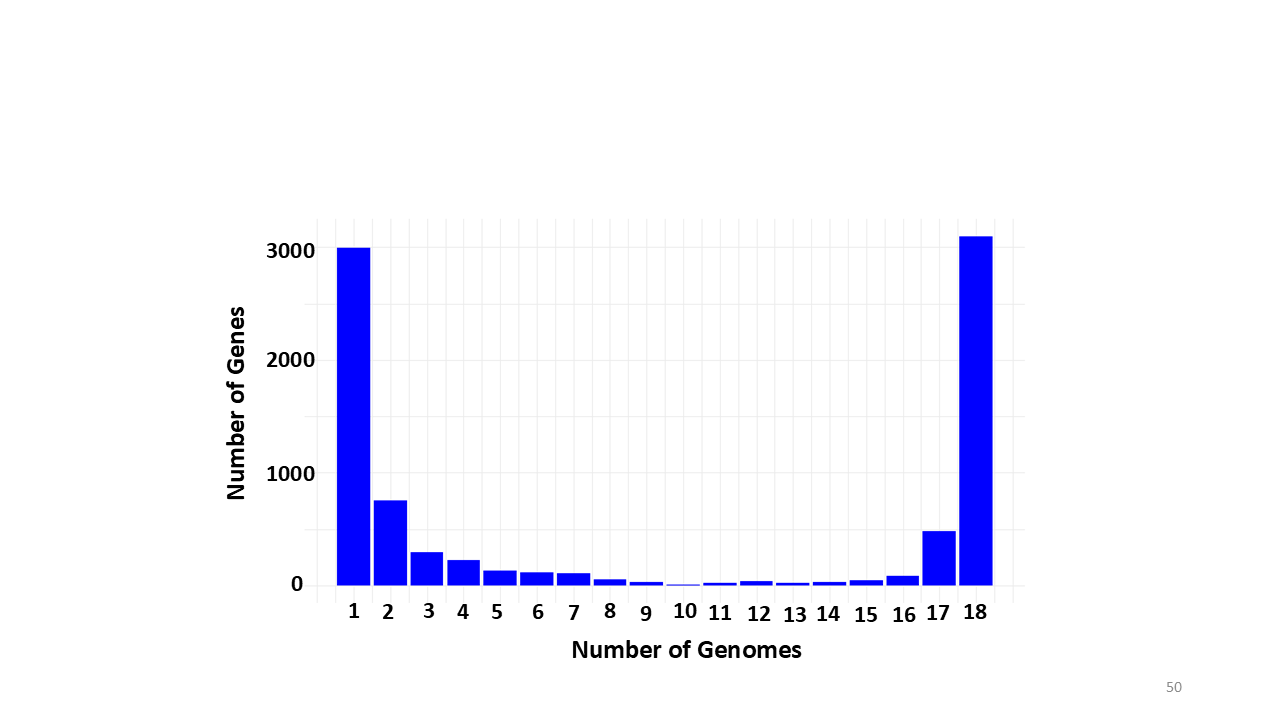
**

**Figure S1: Frequency of genes within the 18 *E. marmotae* genome pan-genome, as analyzed by Roary analysis. At the extreme left end of the x-axis (bar 1) are 2,996 genes present in a single genome and thus regarded as strain-specific genes or possibly pseudogenes. At the right end (bar 18) are genes found in at least one copy in all 18 genomes.**


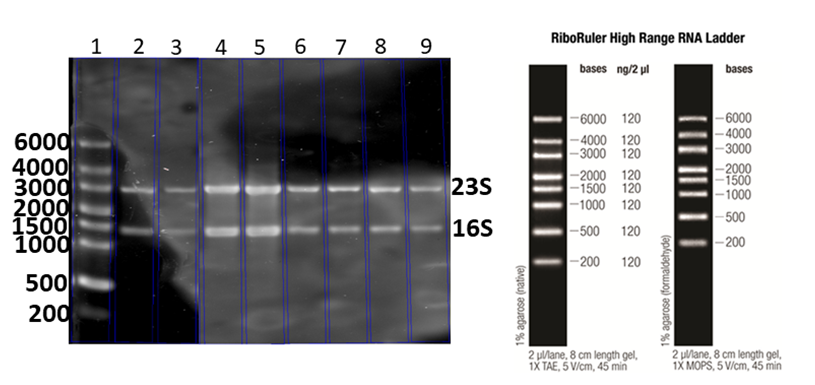


**Figure S2: Representative electrophoresis of RNA samples in 1.5% (w/v) agarose gel, stained with SYBR Safe (Invitrogen) and visualized under UV. Lane 1- RiboRuler High range RNA ladder (Thermo Scientific), while lanes 2-9 consist of the RNA isolated from *E. marmotae* strains incubated at different temperatures. The 23S and 16S rRNA bands indicate good RNA integrity.**


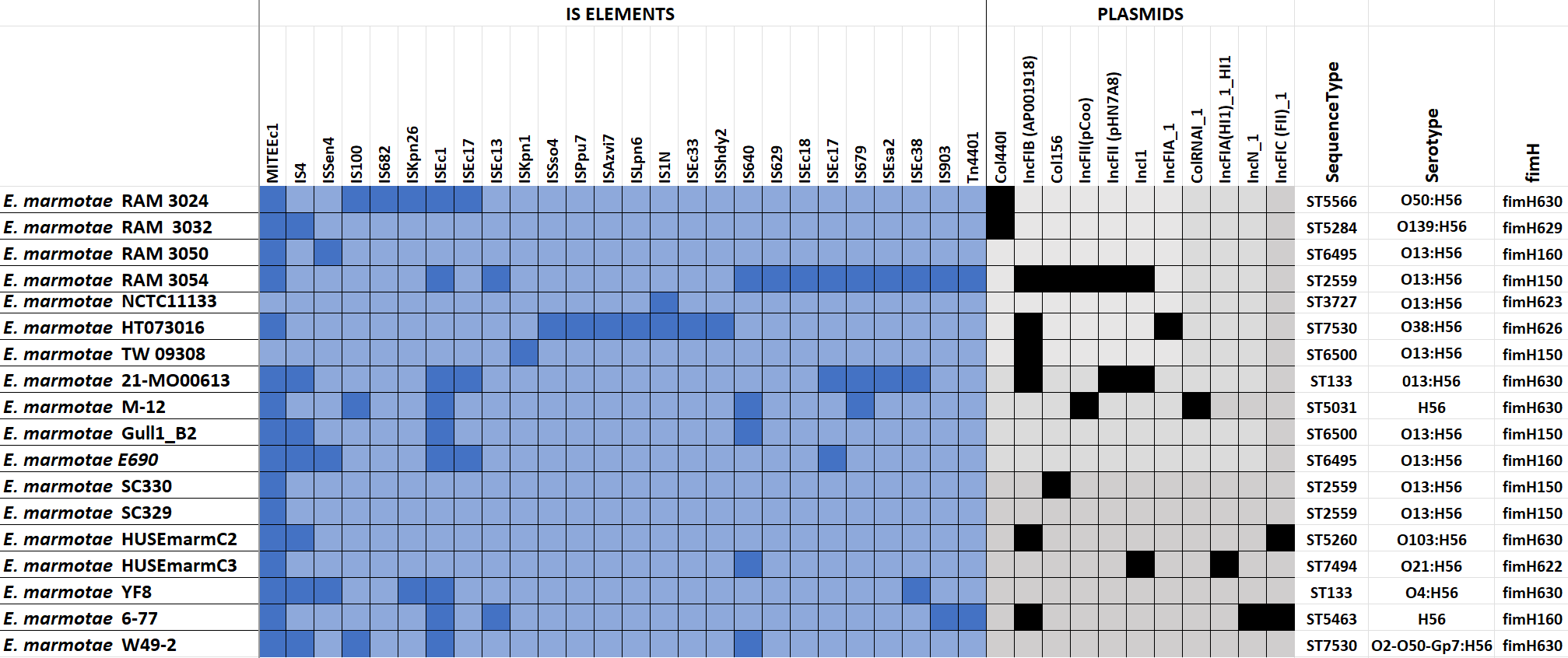


**Figure S3 : Plasmid, insertion elements, serotype, sequence type and FimHtype in 18 strains of *E. marmotae***


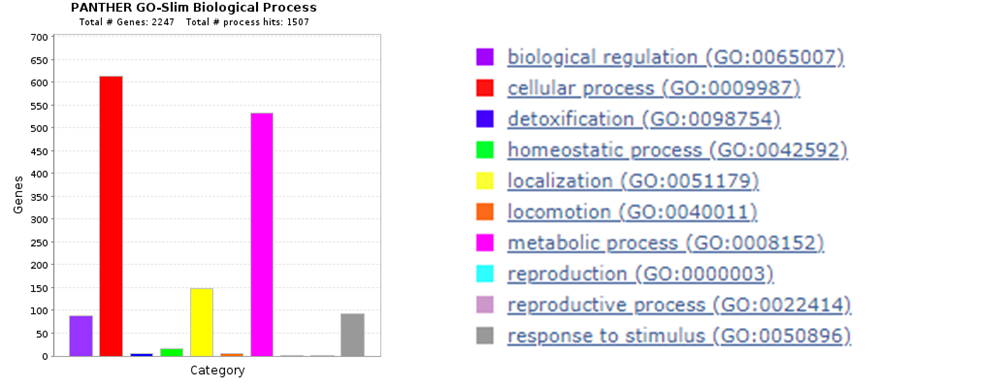
**A**

**
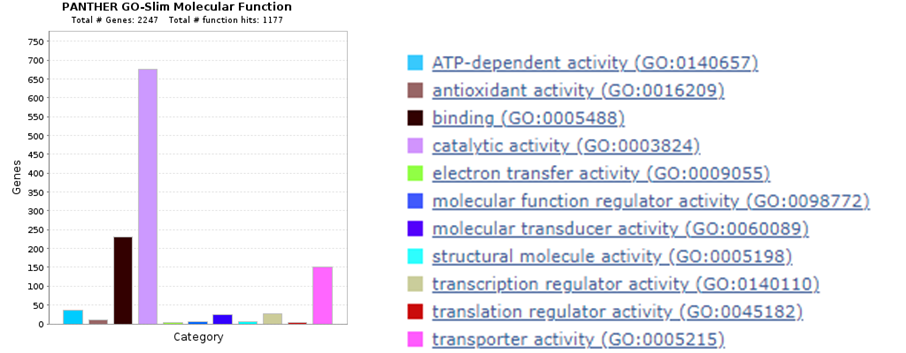
B**

**
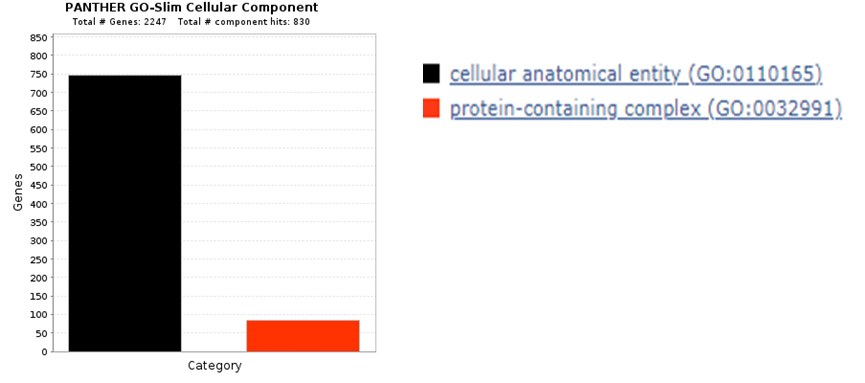
**

**C**

**Figure S4:** **Functional categorization of genes with non-synonymous single nucleotide variants using PANTHER GO-Slim classification. Genes with non-synonymous variants (n=2,247) were analyzed and classified into three Gene Ontology (GO) domains: Molecular function, Biological Process and Cellular component.**


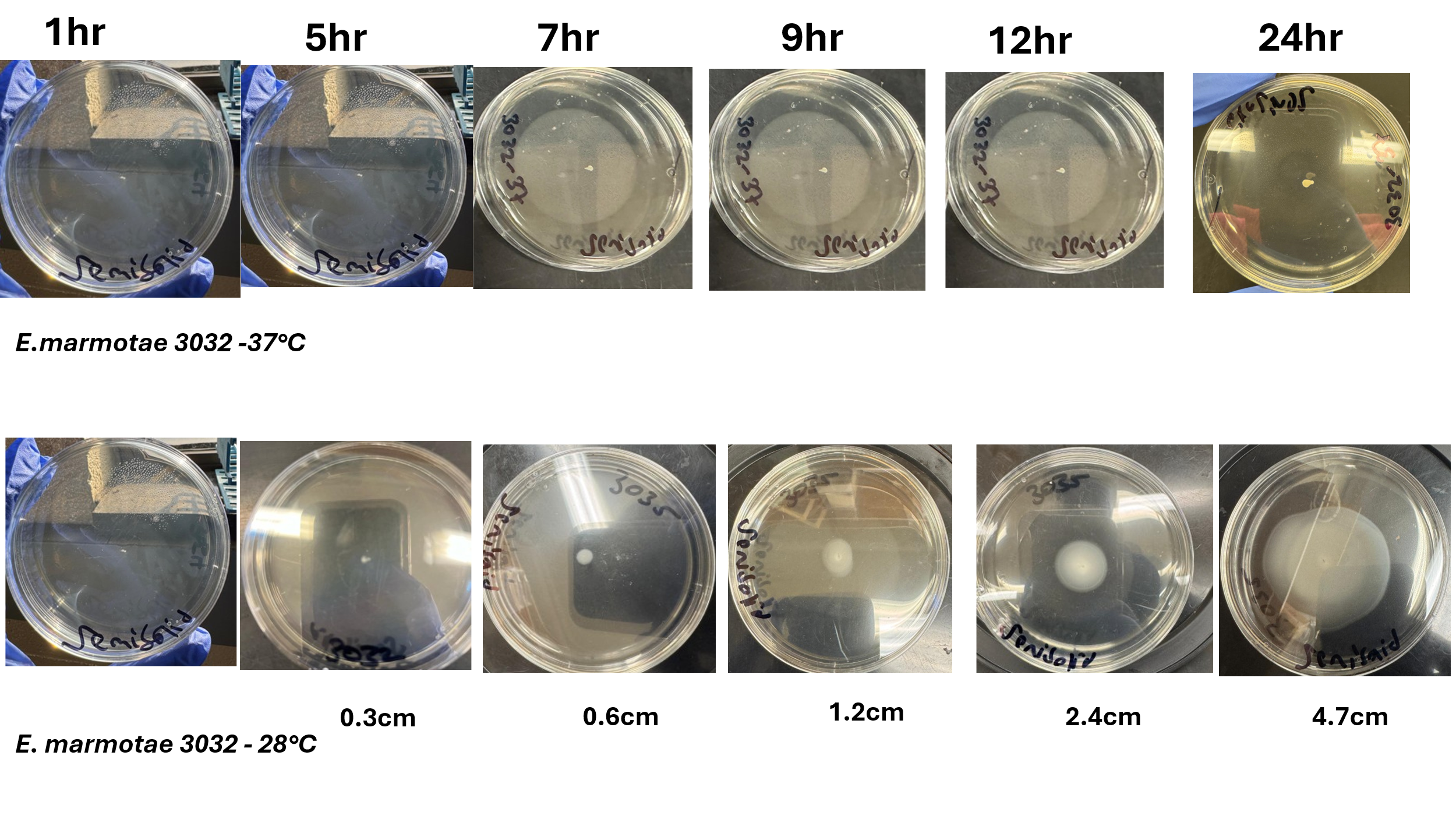


**Figure S5A: Time-course of motility in representative *E. marmotae* (strain 3032) at 37 °C and 28 °C over 24 h. Plates were photographed at the indicated time points. At 37 °C, no visible motility was observed throughout the 24 h period. At 28 °C, progressive outward migration from the inoculation point was evident,**

**
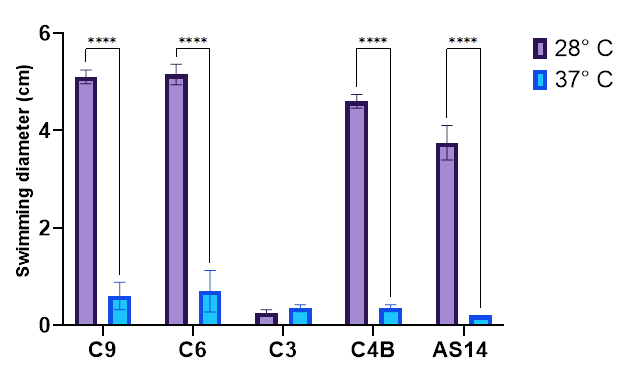
**

**Figure S5B: Motility determined by swimming zone diameters of clinical strains of *E. marmotae* after 24 hr at 37°C and 28°C. The vertical bars represent the standard deviation. Motility at 28°C is significantly greater than at 37°C ****P< 0.0001; two way ANOVA, followed by Sidak’s multiple comparison tests.**


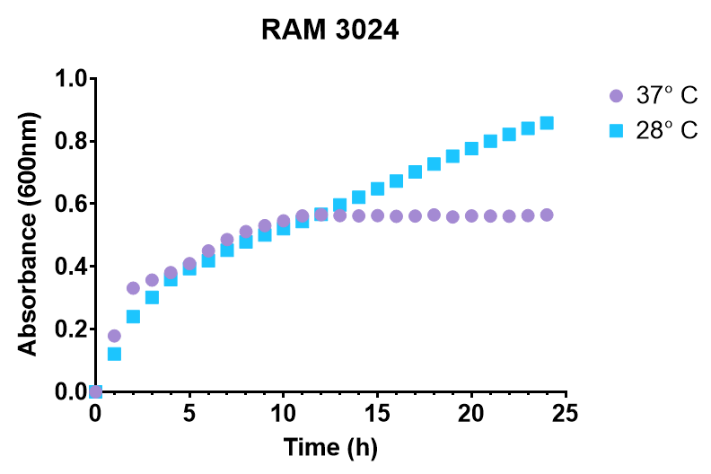


**A**

**B**

**
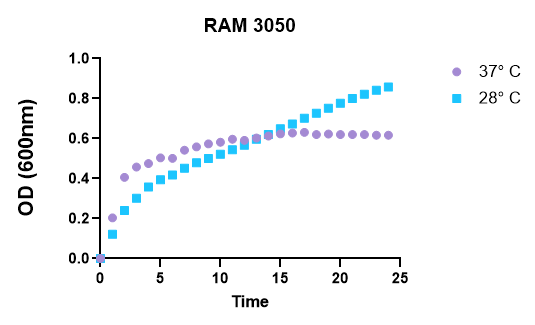
**


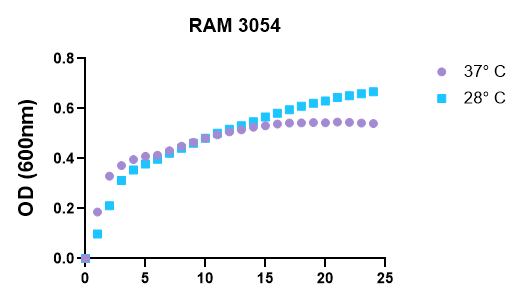


**C**


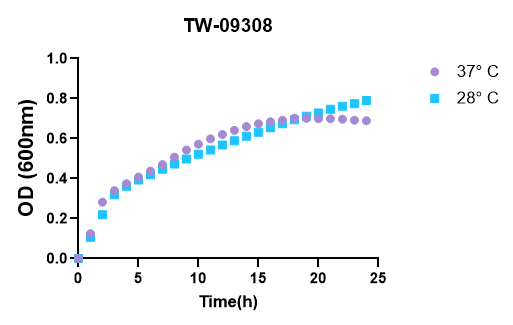


**D**

**E**


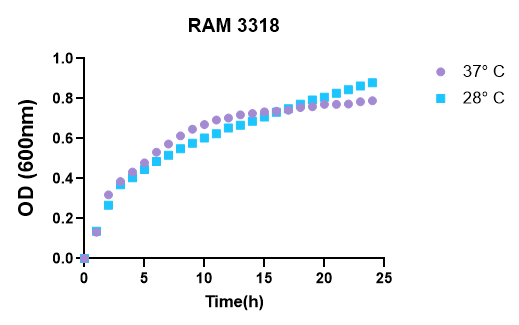


**
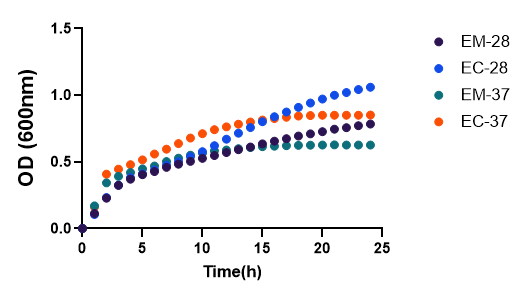
**

**F**

**G**

**Figure S6: A-E Growth curve of *E. marmotae* strains (Ram 3054; Ram 3024; Ram 3050, Ram 3318 and TW 09308) at 37°C and 28°C over the period of 24 hours. F. Growth curve of *E. marmotae* in LB broth at** **37°C and at 28°C over the period of 24 hours in comparison with *E. coli* .**


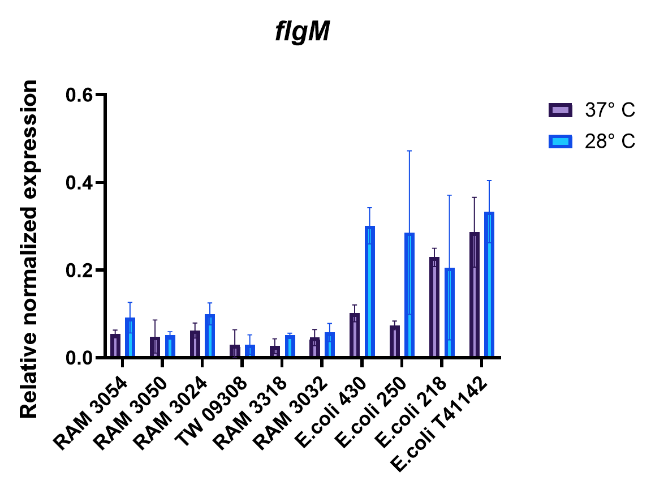

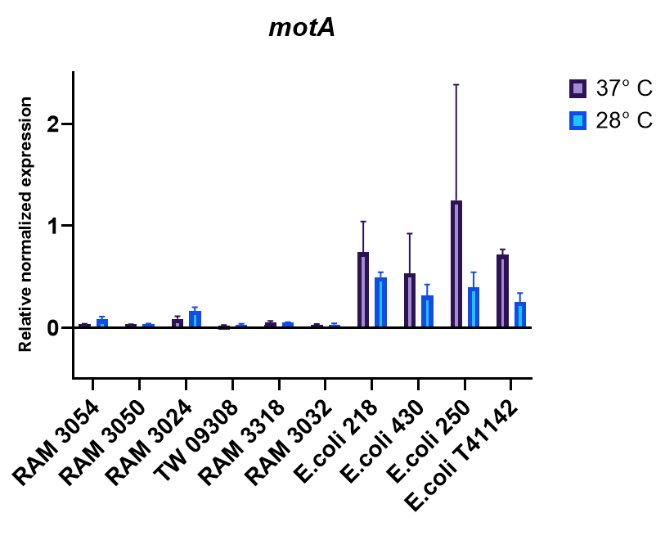

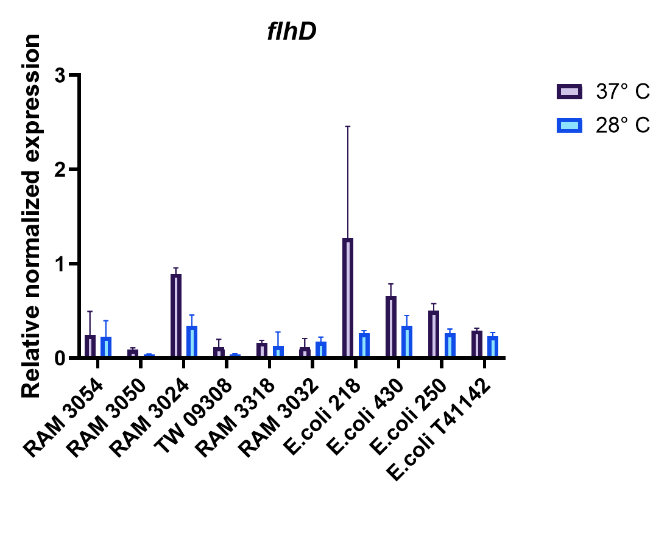

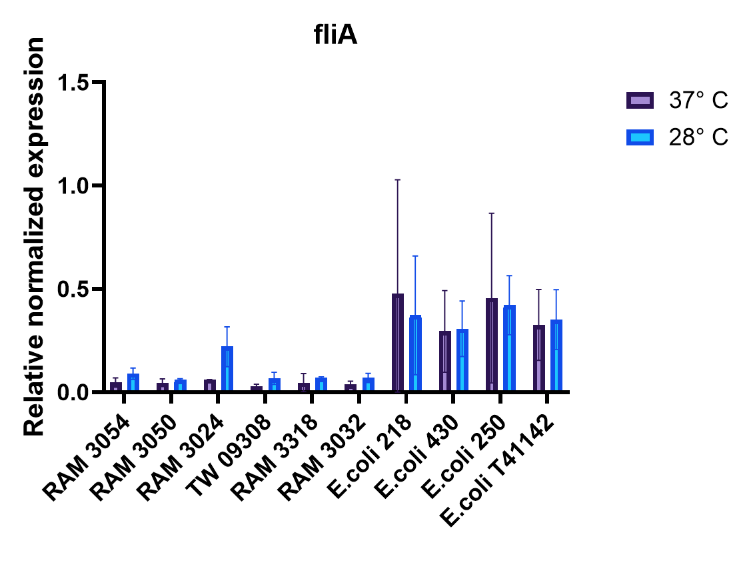


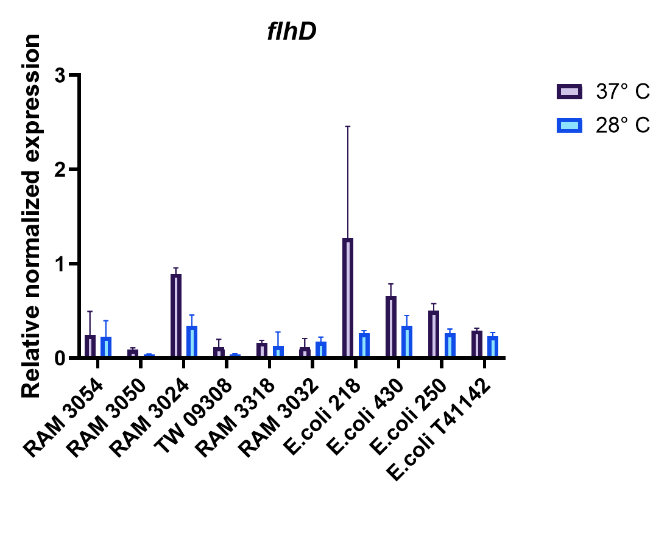

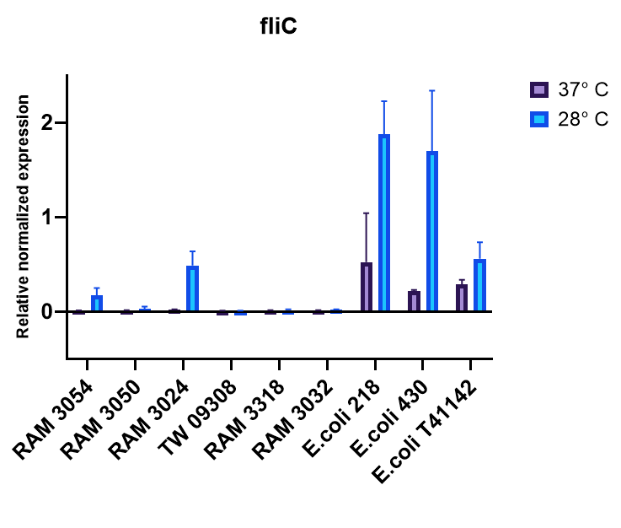


**Figure S7: qRT-PCR relative expression levels of *fliA, motA* and *flhD* in eight *E. marmotae* strains and three *E.coli* strains that were cultured in 37°C and 28°C for 18 h. *adk* gene was used as a reference for the calculation of relative expression levels, using the 2^-△Ct^ method. To normalize distributions, data were transformed with Y = Log(Y) and then analyzed with two-way ANOVA with Sidak’s multiple comparison test to compare each *E. marmotae* and *E. coli* strains at both temperature conditions.**

**
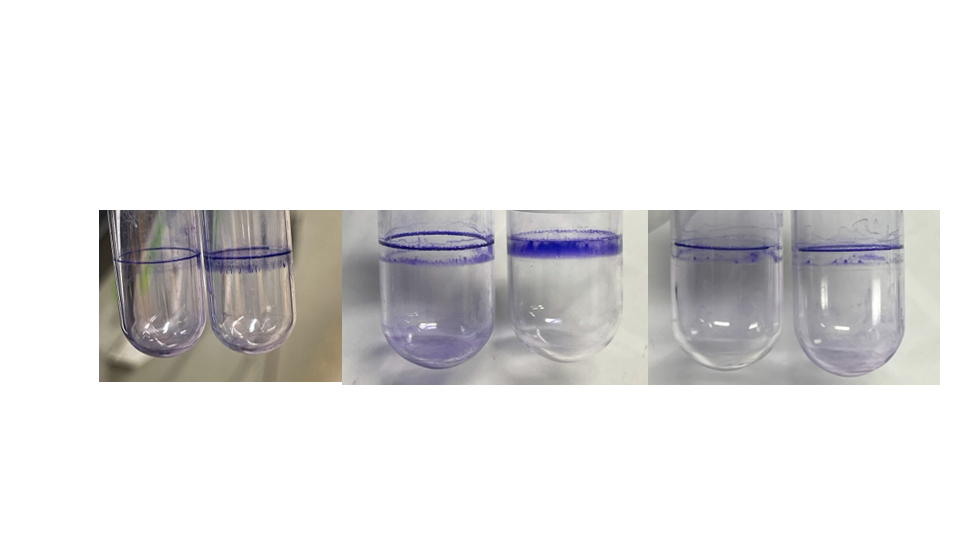
**

**A**

**
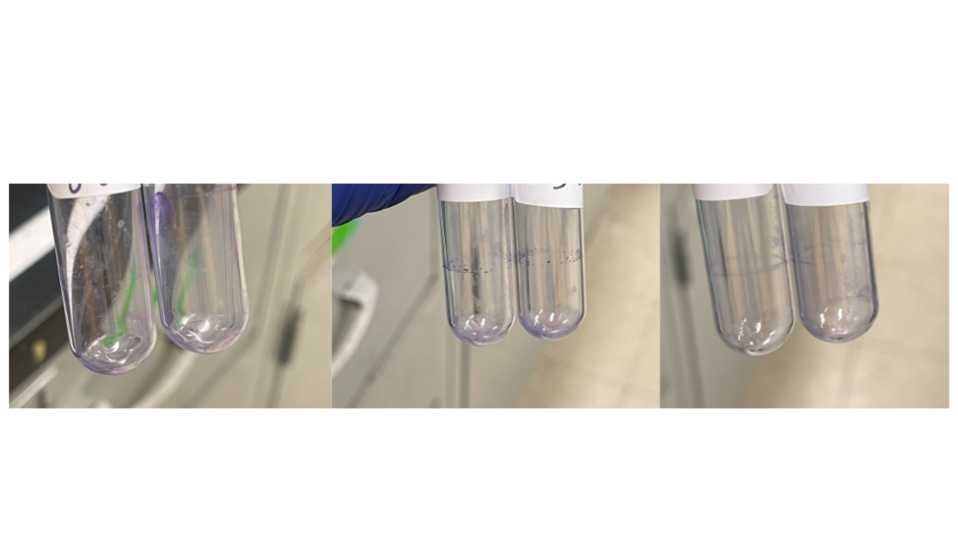
**

**B**

**Figure S8: Biofilm formation of *E. marmotae* in LB Broth for 48h at 28°C (A) and 37°C (B). The biofilm was stained with 0.1% crystal violet and washed with PBS. Biofilm formation is shown with the presence of ring formation at the air-liquid interface.**

**
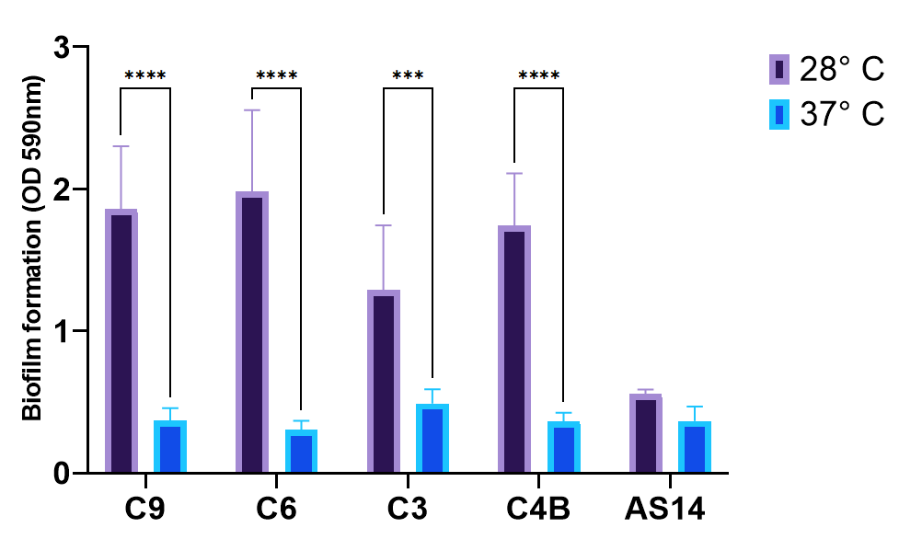
**

**Figure S9: Biofilm formation of clinical strains of *E. marmotae* in LB Broth for 48h at 37°C and 28 °C. The biofilm was stained with 0.1% crystal violet, extracted in 30% acetic acid, and the OD590nm was measured. The bars and error bars represent the mean + standard deviation. *P<0.05, **P<0.01, ***P<0.001, ****P<0.0001; two-way ANOVA followed by Sidak’s multiple comparison**


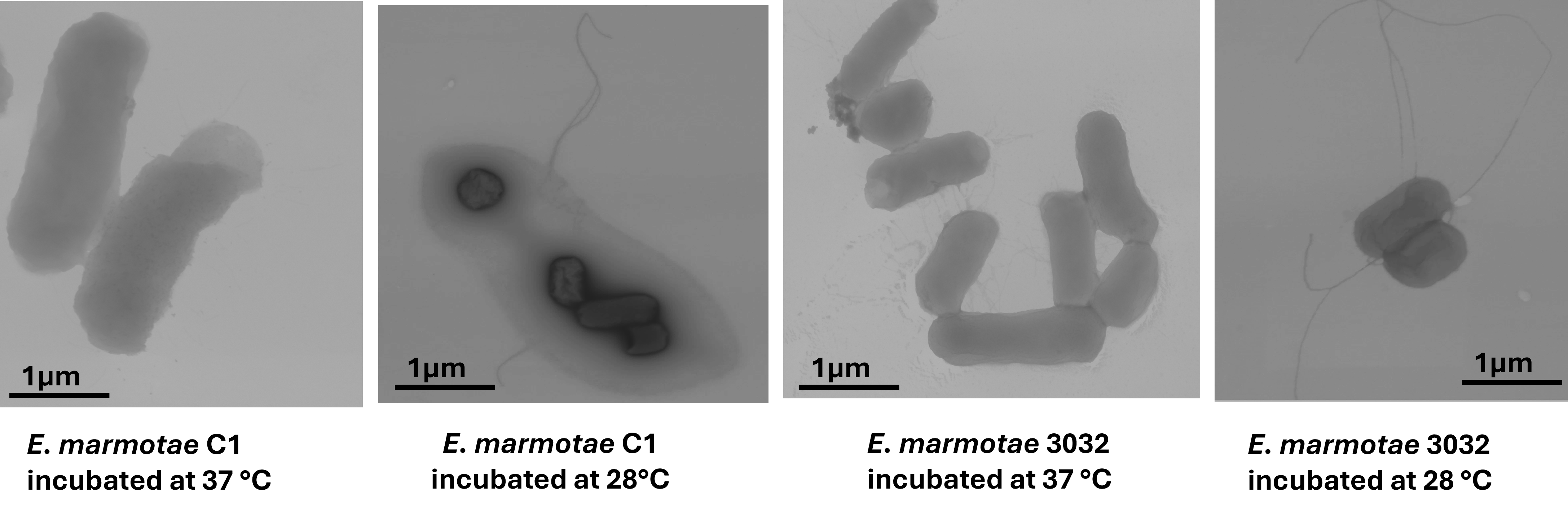


**Figure S10: Negative stain Transmission Electron Microscopy images show flagella present in *E.marmotae* at 28°C but absent from *E. marmotae* at 37°C.**
